# Mapping individual molecular connectomes in Alzheimer's disease

**DOI:** 10.1002/alz.71310

**Published:** 2026-03-26

**Authors:** Zhilei Xu, Mite Mijalkov, Jiawei Sun, Yu‐Wei Chang, Arianna Sala, Giovanni Volpe, Mario Severino, Mattia Veronese, Sara Garcia‐Ptacek, Joana B. Pereira, for the Alzheimer's Disease Neuroimaging Initiative

**Affiliations:** ^1^ Department of Clinical Neuroscience Division of Neuro Karolinska Institutet Solna Sweden; ^2^ Department of Physics University of Gothenburg Gothenburg Sweden; ^3^ Coma Science Group GIGA Consciousness University of Liege Liege Belgium; ^4^ Centre du Cerveau^2^ University Hospital of Liege Liege Belgium; ^5^ Department of Information Engineering University of Padua Padua Italy; ^6^ Department of Neuroimaging King's College London Strand London UK; ^7^ Department of Neurobiology, Division of Clinical Geriatrics Care Sciences and Society, Karolinska Institutet Stockholm Sweden; ^8^ Theme Inflammation and Aging Karolinska University Hospital Solna Sweden

**Keywords:** amyloid, molecular connectome, positron emission tomography, tau, transcriptome

## Abstract

**INTRODUCTION:**

Mapping individual differences is crucial to improve personalized medicine approaches in Alzheimer's disease (AD), which is characterized by strong inter‐individual variability in the accumulation patterns of tau and amyloid beta pathology.

**METHODS:**

We assess the progression of AD across the disease continuum by building individual molecular connectomes using longitudinal positron emission tomography (PET) data.

**RESULTS:**

We demonstrate that these connectomes constitute a unique fingerprint, capable of identifying a single individual from a large group of subjects. Alterations in the connectomes discriminate different diagnostic groups and predict cognitive decline to a higher extent than conventional PET measures. We introduce a novel gene‐specific transcription network analysis that linked individual tau and amyloid connectomes to a common transcriptomic profile of apoptosis, with the tau connectome being specifically related to pyrimidine metabolism, and the amyloid connectome to histone acetylation.

**DISCUSSION:**

Individual molecular connectome mapping provides a novel and sensitive framework to monitor AD progression.

## Highlights

- Individual molecular connectomes constitute a unique fingerprint, capable of identifying a single individual from a large group of subjects.
- Alterations in individual molecular connectomes significantly increase both across the Alzheimer's disease (AD) continuum and over time.
- Alterations in individual molecular connectomes discriminate different diagnostic groups and predict cognitive decline to a higher extent than conventional positron emission tomography measures.
- Susceptibilities of individual tau and amyloid connectomes to AD are linked to a common transcriptomic profile of apoptosis, with the tau connectome being specifically related to pyrimidine metabolism, and the amyloid connectome to histone acetylation.

## 1 BACKGROUND

Alzheimer's disease (AD) is a neurodegenerative disorder characterized by the pathological accumulation of toxic proteins in the brain such as amyloid beta (Aβ) into plaques and tau into neurofibrillary tangles.^1^, ^2^ Recent advances in positron emission tomography (PET) have enabled measuring these two proteinopathies in vivo,^3^, ^4^ revealing strong correlations between PET and *post mortem* Aβ and tau findings.^5^, ^6^, ^7^ Consequently, amyloid PET and tau PET have become important tools in both AD research^8^, ^9^, ^10^ and clinical practice,^11^, ^12^ being widely used to monitor disease progression.^13^, ^14^

However, most studies have assessed Aβ and tau PET burden within groups of predefined brain areas,^5^, ^6^, ^7^, ^8^, ^9^, ^10^, ^11^, ^12^, ^13^, ^14^ such as the ones proposed by the Braak staging model of tau pathology^2^ or by more recently developed data‐driven staging models.^9^, ^10^ This ignores the strong inter‐individual variability that exists in the patterns of Aβ and tau pathology^15^ and their relationships across different brain regions,^16^, ^17^ which challenges these predefined staging models.^18^ While several studies have tried to fit such variations into different subtypes of late onset AD,^19^, ^20^ which is the most frequent form of the disorder, there is no consensus regarding the number of subtypes and whether they are driven by distinct disease mechanisms or basic population variation,^21^ with some researchers even arguing that the variations in Aβ and tau deposition are more continuous across individuals rather than fitting into discrete categories.^22^

This recognition of extensive inter‐individual variation in Aβ and tau pathology has led to several studies investigating these differences, which were found to be shaped by large‐scale brain networks.^23^, ^24^ This aligns with the success of connectome‐based modeling of proteinopathies, also referred to as molecular connectome mapping,^25^ in characterizing neurodegenerative pathology.^26^ However, current molecular connectome mapping approaches are predominantly limited to group‐level analyses,^25^, ^26^ leaving a significant gap in personalized methods that can capture the unique regional relationships of Aβ and tau burden for each individual. Establishing such an approach is crucial for addressing the substantial variability observed across patients with AD and could ultimately improve clinical decision making by enabling more precise diagnoses and personalized treatment strategies.

To address this important issue, here we characterize the individual variations in Aβ and tau pathology across the AD continuum by building individual molecular connectomes that capture the unique variations in Aβ and tau pathology in every patient with respect to the same reference sample. We hypothesize that these molecular connectomes are highly specific to each subject, and can therefore identify single individuals in large samples with very high accuracy, serving as enhanced markers to discriminate different diagnostic groups across the AD continuum. Furthermore, we compare these connectomes to predefined staging models in addition to more recent data‐driven staging models of Aβ and tau pathology to assess whether they show a higher power to discriminate different diagnostic groups and predict decline in various cognitive domains that deteriorate during AD, providing new PET measures for research and clinical purposes. Finally, we investigate the association between individual molecular connectomes and transcriptomic profiles using a novel gene‐specific transcription network approach that uncovers genetic markers linked to these pathologies.

## 2 METHODS

### 2.1 Study design

The primary goal of this study was to examine the sensitivity of individual molecular connectomes in tracking AD progression and their superiority over commonly used PET measures. We first constructed individual molecular connectomes using longitudinal tau PET and amyloid PET data. Next, these connectomes were fed into a connectome fingerprinting analysis to examine its feasibility of investigation at a personalized level. Then, we inspected how these connectomes change across the AD continuum and over time. After that, these connectomes were compared to both traditional and data‐driven PET measures to verify their superiority in tracking disease progression across the AD continuum. Finally, we conducted a connectome–transcriptome association to explore if the susceptibilities of these connectomes to AD is related to specific transcriptional profiles.

**RESEARCH IN CONTEXT**

1. **Systematic review**: The authors reviewed the literature on molecular connectome using traditional sources including PubMed. Current molecular connectome mapping approaches are predominantly limited to group‐level analyses, leaving a significant gap in personalized methods that can capture the unique regional relationships of amyloid beta (Aβ) and tau burden for each individual.
2. **Interpretation**: Our study fills this gap by building individual molecular connectomes that capture the unique variations in Aβ and tau pathology in every patient with respect to the same reference sample. Our findings demonstrated individual molecular connectome mapping of significant sensitivity and superiority in monitoring Alzheimer's disease (AD) progression.
3. **Future directions**: Individual molecular connectome mapping should be implemented in future personalized medicine strategies and testing the effectiveness of current clinical trials in AD.

## 3 DATASETS

Data used in the preparation of this article were obtained from the Alzheimer's Disease Neuroimaging Initiative (ADNI) and the Harvard Aging Brain Study (HABS).

### 3.1 ADNI cohort

The ADNI was launched in 2003 as a public–private partnership, led by Principal Investigator Michael W. Weiner, MD. The primary goal of ADNI has been to test whether serial magnetic resonance imaging, PET, other biological markers, and clinical and neuropsychological assessment can be combined to measure the progression of mild cognitive impairment (MCI) and early AD.^27^ For up‐to‐date information, see www.adni‐info.org. Data used in this study were downloaded from the ADNI data portal (https://adni.loni.usc.edu/) on December 1, 2023, and consisted of 1618 ^18^F‐flortaucipir tau PET scans and 3174 ^18^F‐florbetapir amyloid PET scans from subjects who were diagnosed as either cognitively normal (CN), MCI, or AD based on Mini‐Mental State Examination (MMSE), Clinical Dementia Rating (CDR) score, memory function, and other criteria.^27^ More details about clinical assessments and diagnoses are provided at https://adni.loni.usc.edu/help‐faqs/adni‐documentation/. These PET scans were processed, quality controlled, and converted to standardized uptake value ratios (SUVR) values of 68 cortical regions in the Desikan–Killiany atlas^28^ by the ADNI PET Core team using the inferior cerebellar gray matter and the whole cerebellum as reference regions for ^18^F‐flortaucipir and ^18^F‐florbetapir PET scans, respectively. More details about PET acquisition and processing methods are provided at https://adni.loni.usc.edu/help‐faqs/adni‐documentation/. We used amyloid positivity categorizations by the composite reference cutoff and re‐normalized amyloid SUVR values by diving the SUVR value of the composite reference region as recommended by the ADNI PET Core team.^29^ The composite reference region is a non‐weighted average of the whole cerebellum, brainstem/pons, and subcortical white matter regions.^29^ For the PET scans that passed quality control before and after processing (Pipeline Quality Control Flag > 0), we included the first tau PET and amyloid PET scans of CN Aβ negative (Aβ^−^) subjects and all PET scans of CN Aβ positive (Aβ^+^), MCI Aβ^+^, and AD Aβ^+^ subjects. The tau PET scans of Aβ^−^ subjects with a diagnosis of MCI and AD were excluded due to mounting evidence showing these individuals are not part of the AD continuum.^30^ Each Aβ^+^ subject included in our analyses had up to five tau PET scans and six amyloid PET scans.

### 3.2 HABS cohort

The HABS was launched in 2010 to elucidate the earliest changes in molecular, functional, and structural imaging markers that signal the transition from normal cognition to progressive cognitive decline along the trajectory of preclinical AD.^31^ Data used in this study were downloaded from the XNAT data portal (https://central.xnat.org/) on June 29, 2023, and consisted of 343 ^18^F‐flortaucipir tau PET scans and 696 ^11^C‐Pittsburgh compound B (PiB) amyloid PET scans from subjects who were diagnosed as either CN, MCI, or AD based on CDR score, cognitive data, and relevant medication history. These PET scans were processed, quality controlled, and converted to SUVR values of 68 cortical regions in the Desikan–Killiany atlas^28^ by the HABS team using the bilateral cerebellum gray matter as reference region for both ^18^F‐flortaucipir and ^11^C‐PiB PET scans. More details about clinical assessments and diagnosis as well as PET acquisition and processing methods are provided at https://habs.mgh.harvard.edu/researchers/data‐details/. We used amyloid positivity categorizations provided by the HABS team and included the first tau PET and amyloid PET scans of CN Aβ^−^ subjects and all PET scans of CN Aβ^+^ subjects. We excluded PET scans of MCI and AD subjects because of the small sample size (MCI: *n* = 16, AD: *n* = 3). Each CN Aβ^+^ subject had up to three tau PET scans and four amyloid PET scans.

Informed written consent was obtained from all participants by each cohort's investigators. All protocols were approved by each cohort's respective institutional ethical review board. Demographic and clinical characteristics of the included participants from ADNI and HABS are provided in Tables 1 and S1 in supporting information. Demographic and clinical characteristics of the included participants with tau PET scans after partial volume correction from ADNI are provided in Table S2 in supporting information.

**TABLE 1:** Demographic and clinical characteristics of the included participants.

	Participants with tau PET scans					Participants with amyloid PET scans				
	CN Aβ^−^	CN Aβ^+^	MCI Aβ^+^	AD Aβ^+^	All	CN Aβ^−^	CN Aβ^+^	MCI Aβ^+^	AD Aβ^+^	All
**ADNI cohort**										
*N*	344	133	142	87	706	388	177	324	252	1141
Scans	344	236	204	125	909	388	313	502	318	1521
Age at first scan (years)	70.8 (50.5–94.2)	73.9 (56.5–91.4)	75.5 (56.0–92.4)	76.9 (55.5–93.9)	73.2 (50.5–94.2)	71.0 (55.8–95.2)	76.3 (56.5–92.4)	75.2 (55.0–96.3)	76.5 (55.3–95.7)	74.6 (55.0–96.3)
Follow‐up scans	–	1 (1–4)	1 (1–3)	1 (1–3)	1 (1–4)	–	1 (1–4)	1 (1–5)	1 (1–2)	1 (1–5)
Follow‐up years	–	2.1 (0.8–5.4)	2.1 (0.6–4.9)	1.4 (0.5–3.0)	2.0 (0.5–5.4)	–	3.6 (1.0–9.5)	2.5 (1.8–11.3)	2.0 (1.0–6.2)	2.3 (1.0–11.3)
Sex (male/female)	150/194	49/84	73/69	51/36	323/383	181/207	67/110	179/145	138/114	565/576
Education (years)	17.0 (11.0–20.0)	16.0 (12.0–20.0)	16.0 (12.0–20.0)	16.0 (10.0–20.0)	16.0 (10.0–20.0)	16.0 (8.0–20.0)	16.0 (6.0–20.0)	16.0 (8.0–20.0)	16.0 (8.0–20.0)	16.0 (6.0–20.0)
MMSE at first scan	29.0 (23.0–30.0)	29.0 (24.0–30.0)	27.0 (19.0–30.0)	23.0 (9.0–30.0)	29.0 (9.0–30.0)	29.0 (24.0–30.0)	29.0 (25.0–30.0)	28.0 (19.0–30.0)	23.0 (7.0–30.0)	28.0 (7.0–30.0)
**HABS cohort**										
*N*	132	63	–	–	195	207	101	–	–	308
Scans	132	102	–	–	234	207	202	–	–	409
Age at first scan (years)	74.0 (64.8–92.8)	76.5 (68.3–91.5)	–	–	75.0 (64.8–92.8)	71.8 (62.8–90.0)	76.0 (65.3–91.5)	–	–	73.8 (62.8–91.5)
Follow‐up scans	–	1 (1–2)	–	–	1 (1–2)	–	1.5 (1–3)	–	–	1.5 (1–3)
Follow‐up years	–	2.1 (1.2–4.9)	–	–	2.1 (1.2–4.9)	–	3.5 (1.6–5.7)	–	–	3.5 (1.6–5.7)
Sex (male/female)	54/78	49/53	–	–	79/116	84/123	89/113	–	–	122/186
Education (years)	16.0 (6.0–20.0)	16.0 (11.0–20.0)	–	–	16.0 (6.0–20.0)	16.0 (6.0–20.0)	16.0 (8.0–20.0)	–	–	16.0 (6.0–20.0)
MMSE at first scan	30.0 (25.0–30.0)	29.0 (26.0–30.0)	–	–	30.0 (25.0–30.0)	29.0 (25.0–30.0)	29.0 (26.0–30.0)	–	–	29.0 (25.0–30.0)

*Notes*: Data presented in the table are reported as median (range), unless otherwise stated. *P* values corresponding to the between‐group statistical comparisons are shown in Table S1 in supporting information.

Abbreviations: Aβ, amyloid beta; AD, Alzheimer's Disease; ADNI, Alzheimer's Disease Neuroimaging Initiative; CN, cognitively normal; HABS, Harvard Aging Brain Study; MCI, mild cognitive impairment; MMSE, Mini‐Mental State Examination; PET, positron emission tomography.

### 3.3 Constructing individual molecular connectomes

**FIGURE 1:**
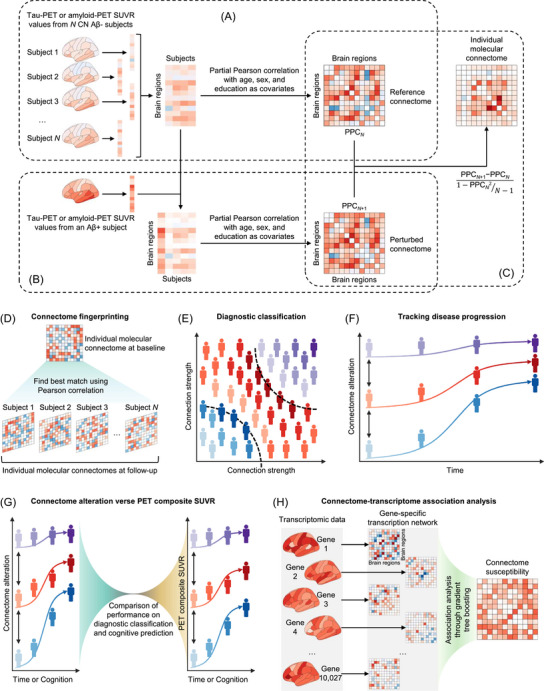
Study design overview. A–C, Overview of constructing individual molecular connectomes. A reference molecular connectome was first constructed across *N* CN Aβ^−^ subjects by computing the partial Pearson correlation coefficient $\mathrm{PP}C_{N}$ between tau PET or amyloid PET SUVR values for each pair of cortical regions while controlling for age, sex, and education (A). Then, a perturbed molecular connectome $\mathrm{PP}C_{N+1}$ was constructed by adding an Aβ^+^ subject to the CN Aβ^−^ group and re‐calculating the partial Pearson correlation coefficient across these *N*+1 subjects (B). The individual molecular connectome of this Aβ^+^ subject was obtained through dividing the difference between the perturbed molecular connectome $\mathrm{PP}C_{N+1}$ and the reference molecular connectome $\mathrm{PP}C_{N}$ by its standard deviation $\frac{1-\mathrm{PP}{C_{N}}^{2}}{N-1}$ (C). D–H, Overview of analysis pipeline. Individual molecular connectomes obtained in (C) were fed into the analyses of connectome fingerprinting (D), diagnostic classification (E), and tracking disease progression (F). Then, these connectomes were compared to commonly used PET composite SUVR values for the performance on diagnostic classification and cognitive prediction (G). Finally, we examined the association between the susceptibility of these connectomes to AD and the brain transcription (H). Aβ, amyloid beta; AD, Alzheimer's disease; CN, cognitively normal; PET, positron emission tomography; SUVR, standardized uptake value ratio.

We constructed individual molecular connectomes separately for ADNI and HABS by adopting a statistical perturbation analysis of a single sample against a group of given reference samples, as described previously.^32^, ^33^, ^34^ As shown in Figure 1A, we first constructed a reference tau connectome $\mathrm{PP}C_{N}$ across *N* CN Aβ^−^ subjects by calculating the partial Pearson correlation coefficient between SUVR values of tau pathology for each pair of the 68 cortical regions defined by the Desikan–Killiany atlas,^28^ while controlling for age, sex, and education using the MATLAB function partialcorr. Then, an Aβ^+^ subject was added to the CN Aβ^−^ group to construct a perturbed tau connectome $\mathrm{PP}C_{N+1}$ by re‐calculating the partial Pearson correlation coefficient across these *N*+1 subjects (Figure 1B). Afterward, we obtained the individual tau connectome of this Aβ^+^ subject by calculating the difference between the perturbed tau connectome $\mathrm{PP}C_{N+1}$ and the reference tau connectome $\mathrm{PP}C_{N}$ and normalized it by dividing it by its standard deviation $\frac{1-\mathrm{PP}{C_{N}}^{2}}{N-1}$ (Figure 1C). For each pair of brain regions *i* and *j*, we evaluated the connection strength as $Strength\;\left(i,j\right)=\frac{\mathrm{PP}C_{N+1}\left(i,\;j\right)-\mathrm{PP}C_{N}\left(i,\;j\right)}{\frac{1-\mathrm{PP}C_{N}{\left(i,j\right)}^{2}}{N-1}}$. The individual amyloid connectome was constructed through the same procedure using the SUVR values of Aβ pathology. To assess whether apolipoprotein E (*APOE*) status had an impact on our findings, we repeated the analysis including *APOE* as an additional covariate. The resulting *APOE*‐adjusted connectomes showed high correspondence with the *APOE*‐unadjusted connectomes (Figure S1 in supporting information), suggesting that including *APOE* status as an additional covariate does not affect the connectivity patterns.

Therefore, we proceeded using only age, sex, and education as covariates. Of note, the sign of the perturbed connection is mathematically independent of that of the reference connection. Therefore, the sign of the connection in an individual molecular connectome only indicates the direction in which the perturbed connection deviates from the reference one, not an increasing or decreasing of the connection strength in that single sample against the group of given reference samples.

### 3.4 Identifying single subjects using individual molecular connectomes

**FIGURE 2:**
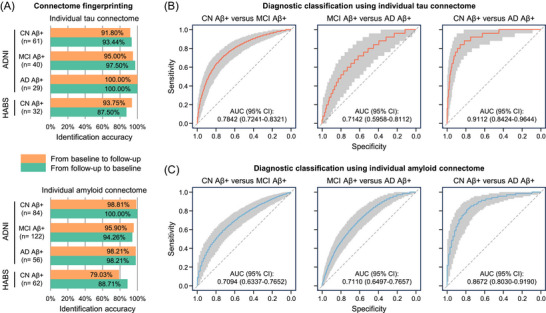
Connectome fingerprinting and diagnostic classification using individual molecular connectomes. A, Connectome fingerprinting accuracy within each diagnostic group. Each bar plot represents a single value. B and C, Results from the ROC curve analysis for binary classification of two diagnostic groups using individual tau (B) and amyloid (C) connectomes. Solid line represents the median ROC curve across 1000 repeated experiments using different training and testing samples. Gray shading represents the 95% CI of the ROC curve across the 1000 repeated experiments. AUC was reported as median (95% CI) across the 1000 repeated experiments. Aβ, amyloid beta; AD, Alzheimer's disease; ADNI, Alzheimer's Disease Neuroimaging Initiative; AUC, area under the receiver operating characteristic curve; CI, confidence interval; CN, cognitively normal; HABS, Harvard Aging Brain Study; MCI, mild cognitive impairment; ROC, receiver operating characteristic.

If individual molecular connectome enables investigation of single subjects, it should be both unique and reliable for each subject, similar to a fingerprint that allows identifying single subjects.^35^ We examined this hypothesis in Aβ^+^ subjects with at least one follow‐up PET scan from ADNI and HABS separately. For Aβ^+^ subjects with more than one follow‐up PET scan, we only included the first follow‐up scan in our analysis. To avoid the potential effect of diagnostic group, we conducted subject identification within each diagnostic group (CN Aβ^+^, MCI Aβ^+^, and AD Aβ^+^) through an iterative process similar to that by Finn et al.^35^ As illustrated in Figure 1D, for each subject at baseline we identified the best matched one from *N* subjects at follow‐up by calculating the Pearson correlation coefficient between the values in the upper triangles of their individual molecular connectome matrices using the MATLAB function corr. If the subject at baseline and the best matched one at follow‐up have the same “identity,” the identification is successful, or failed, if they have different identities. In this context, an individual's “identity” (Figure S2 in supporting information) refers to their unique molecular connectivity profile, defined by the pattern of pairwise connections represented in their connectome matrix, both for amyloid and tau PET connectomes. Thus, the fingerprinting tests whether each individual's connectivity profile at baseline is more similar to its own profile across follow‐up scans than to the profiles of other participants.^35^, ^36^ This identification procedure was repeated for each subject at baseline and reversed in the direction from follow‐up to baseline as shown in Figure 2A. We computed the identification accuracy rate by dividing the count of successful identifications by the count of all identifications.

### 3.5 Discriminating different diagnostic groups using individual molecular connectomes

After demonstrating the plausibility for investigation of single subjects, we examined whether individual molecular connectomes could discriminate different diagnostic groups across the AD continuum using the ADNI cohort as illustrated in Figure 1E. For the 565 individual tau connectomes, we randomly divided them into a training dataset including 100 connectomes from each of the CN Aβ^+^, MCI Aβ^+^, and AD Aβ^+^ group and a testing dataset including the remaining 265 connectomes. For the 1133 individual amyloid connectomes, we randomly divided them into a training dataset including 250 connectomes from each of the CN Aβ^+^, MCI Aβ^+^, and AD Aβ^+^ group and a testing dataset including the remaining 383 connectomes. The 300 tau training connectomes and the 750 amyloid training connectomes were separately fed into a gradient tree boosting model to train three binary classifiers (CN Aβ^+^ vs. MCI Aβ^+^, MCI Aβ^+^ vs. AD Aβ^+^, and CN Aβ^+^ vs. AD Aβ^+^). The optimal classifiers were tested using the 265 tau testing connectomes and the 383 amyloid testing connectomes, respectively. The accuracy rates of the above binary classifiers were stably estimated by repeating the randomly selecting training connectomes and classifier training and testing procedures 1000 times. We examined the effectiveness of these classifiers by comparing their area under the receiver operating characteristic curve across these 1000 repetitions to the chance level of 0.5 using one‐sided sign tests and correcting for false discovery rate (FDR).

### 3.6 Examining how the individual molecular connectomes changed across the AD continuum and over time

**FIGURE 3:**
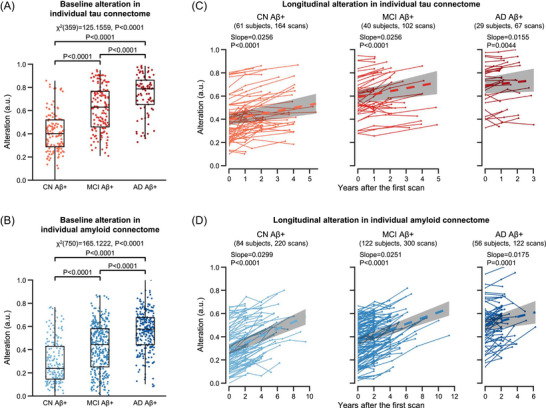
Alterations in individual molecular connectomes increase across the AD continuum and over time. Baseline alterations in individual tau (A) and amyloid (B) connectomes across the AD continuum. Each dot represents one subject. Box plots depict the interquartile range and the median value of the distribution. Whiskers extend to the nearest data points ± 1.5 times the interquartile range. The effects of diagnostic group (CN Aβ^+^, MCI Aβ^+^, and AD Aβ^+^) on individual molecular connectomes were examined by Kruskal–Wallis test followed by post hoc Wilcoxon rank‐sum tests. The significance levels of the Kruskal–Wallis test and post hoc Wilcoxon rank‐sum tests were evaluated through 10,000 permutations and were corrected for FDR. Longitudinal alterations in individual tau (C) and amyloid (D) connectomes across the AD continuum. Each dot represents one PET scan. Each thin line connects PET scans from one subject. Bold dashed line and gray shading represent the fitted line and its 95% confidence intervals from the linear mixed‐effects model. *P* values were extracted from the linear mixed‐effects model and were corrected for FDR. Aβ, amyloid beta; AD, Alzheimer's disease; a.u., arbitrary unit; CN, cognitively normal; FDR, false discovery rate; MCI, mild cognitive impairment; PET, positron emission tomography.

For each Aβ^+^ subject's individual tau or amyloid connectome from ADNI, we evaluated its extent of alteration from those of CN Aβ^−^ subjects using a single value that combines each connection's strength with its contribution to discriminating different diagnostic groups. To evaluate each connection's aggregate contribution to discriminating the three diagnostic groups, we fed the same 300 tau training connectomes and 750 amyloid training connectomes as described above separately into a gradient tree boosting model to train a multiclass classifier. Each connection's contribution to discriminating the three diagnostic groups was determined by the gradient tree boosting model during the training procedure. We repeated the randomly selected training connectomes and classifier training procedures 1000 times. Each connection's aggregate contribution was stably estimated by averaging across these 1000 repetitions and is provided in Figures S3 and S4 in supporting information. The connectome alteration extent was computed as: $\mathrm{Alteration}\;=\;\mathrm{BoxCox}(\sqrt{\sum \mathrm{Strengt}{h_{i}}^{2}\times \mathrm{Contributio}n_{i}})$. $\mathrm{Strengt}h_{i}$ and $\mathrm{Contributio}n_{i}$ indicate the strength and the contribution of the ith connection, respectively. BoxCox indicates a Box–Cox transformation of data toward normality that was implemented using the MATLAB function boxcox. For illustration purposes, we normalized the alteration extent across all tau or amyloid connectomes of Aβ^+^ subjects to the range of [0, 1] using the MATLAB function normalize as shown in Figure 3. A larger value indicates a greater alteration extent.

We first compared the extent of connectome alteration at baseline among the three diagnostic groups using Kruskal–Wallis test followed by post hoc one‐sided Wilcoxon rank‐sum tests. The significance levels of the Kruskal–Wallis test and post hoc Wilcoxon rank‐sum tests were evaluated through 10,000 permutations and were corrected for FDR. Then, we examined the alterations in individual molecular connectomes over time by fitting a linear mixed‐effects model. The linear mixed‐effects model was first fitted within each diagnostic group using the extent of connectome alteration as the outcome and the time (years after the first PET scan) as predictors, while controlling for individual‐specific random intercepts and slopes. These models included all follow‐up scans for all participants, and no scans were excluded based on time differences between their follow‐up and baseline measurements. The fitted line and its 95% confidence intervals as well as its slope's significance level were extracted from the MATLAB function fitlme. The significance level of the slope was corrected for FDR. We also fitted the linear mixed‐effects model across diagnostic groups by adding the diagnostic group and its interaction with time as predictors.

To examine the robustness of our results, we repeated the above analyses using tau PET data after partial volume correction from ADNI and validated our findings in the HABS cohort. The contribution matrices shown in Figures S3 and S4 were used throughout these analyses to evaluate the extent of connectome alteration.

### 3.7 Comparing the alterations in individual molecular connectomes to previous methods in discriminating different diagnostic groups

We first measured SUVR values using the ADNI cohort for four sets of composite regions defined by previous studies. For ^18^F‐flortaucipir tau PET scans, we calculated volume‐weighted average SUVR values for two sets of previously defined composite regions. The first one includes three composite regions corresponding to Braak stages I to II, III to IV, and V to VI, as defined by Cho et al.^8^ The second includes five composite regions corresponding to five data‐driven tau stages reported by Leuzy et al.^9^ For ^18^F‐florbetapir amyloid PET scans, we calculated volume‐weighted average SUVR value for two sets of previously defined composite regions. The first one includes a global composite region that includes the frontal, temporal, parietal, occipital, anterior cingulate, posterior cingulate, and precuneus cortices, as defined by Wong et al.^3^ The second includes four composite regions corresponding to four data‐driven amyloid stages reported by Grothe et al.^10^ The label for these four sets of composite regions is provided in Table S3 in supporting information.

Next, we compared the performance of alterations in individual molecular connectomes to composite SUVR values in discriminating different diagnostic groups. For the 565 ^18^F‐flortaucipir tau PET scans, we divided them into a training dataset including 100 scans from each of the CN Aβ^+^, MCI Aβ^+^, and AD Aβ^+^ group and a testing dataset including the remaining 265 scans. For the 1133 ^18^F‐florbetapir amyloid PET scans, we randomly divided them into a training dataset including 250 scans from each of the CN Aβ^+^, MCI Aβ^+^, and AD Aβ^+^ group and a testing dataset including the remaining 383 scans. The connectome alteration extent and each of the composite SUVR values of the 300 tau PET training scans and the 750 amyloid PET training scans were separately fed into a gradient tree boosting model to train three binary classifiers (CN Aβ^+^ vs. MCI Aβ^+^, MCI Aβ^+^ vs. AD Aβ^+^, and CN Aβ^+^ vs. AD Aβ^+^). The optimal classifiers were tested using the 265 tau PET testing scans and the 383 amyloid PET testing scans, respectively. The accuracy rate of the above gradient tree boosting classifiers were stably estimated by repeating the randomly selected training scans and classifier training and testing procedures 1000 times. We compared the discriminative accuracy of models using connectome alteration extent to those using composite SUVR values through one‐sided paired *t* tests across these 1000 repetitions. The significance levels of these paired *t* tests were evaluated through 10,000 permutations and corrected for FDR.

### 3.8 Comparing the alterations in individual molecular connectomes to previous methods in predicting longitudinal cognitive decline

Then, we compared the performance of alterations in individual molecular connectomes to composite SUVR values in detecting longitudinal cognitive decline using the ADNI cohort. For each of the 565 ^18^F‐flortaucipir tau PET and 1133 ^18^F‐florbetapir amyloid PET scans from Aβ^+^ subjects, we matched it to the nearest cognitive examination that was conducted within 6 months of it. The examination assessed the performance of global cognition (13‐item Alzheimer's Disease Assessment Scale Cognitive subscale [ADAS13]) and specific cognitive domains, including visuospatial function (CLOCKSCOR in Clock Drawing), memory (Q4SCORE in ADAS [ADAS‐Q4]), attention (TRAASCOR in Trail Making Test—Part A), and executive function (TRABSCOR in Trail Making Test—Part B). Detailed calculation of these cognitive outcomes is provided at https://adni.loni.usc.edu/wp‐content/themes/freshnews‐dev‐v2/documents/clinical/ADNI‐1_Protocol.pdf . If any measurement of these five cognitive performances was unavailable, we treated it as a missing value in the following analysis.

For tau PET, we included the 362 baseline scans as a training dataset and the 203 follow‐up scans as a testing dataset. We trained a benchmark predictive model based on gradient tree boosting for each of the five cognitive performances using age, sex, education, and dementia status corresponding to these 565 tau PET scans. The alteration extent of individual tau connectome and each of the eight tau PET composite SUVR values were separately added into the benchmark predictive model. For amyloid PET, we included the 753 baseline scans as a training dataset and the 380 follow‐up scans as a testing dataset. We trained a benchmark predictive model based on gradient tree boosting for each of the five cognitive performances using age, sex, education, and dementia status corresponding to these 1133 amyloid PET scans. The alteration extent of individual amyloid connectome and the five amyloid PET composite SUVR values were separately added to the benchmark predictive model. We examined the performance of these predictive models by calculating Pearson correlation coefficient between predicted values and true values. We compared the performance of the benchmark predictive model to that of other models using one‐sided paired *t* tests on the Pearson correlation coefficient. The significance levels of these paired *t* tests were evaluated through 10,000 permutations and were corrected for FDR.

### 3.9 Association analysis between individual molecular connectomes and the brain's transcriptome

To examine the potential genetic contribution to the susceptibilities of individual molecular connectomes to AD, we performed a connectome–transcriptome association analysis using the Allen Human Brain Atlas (AHBA) dataset.^37^ First, we estimated the susceptibilities of individual tau and amyloid connectomes to AD by calculating the common logarithm of each connection's contribution to discriminating different diagnostic groups shown in Figures S3 and S4, respectively.

Next, we constructed a gene‐specific transcription network using the AHBA dataset by adopting a statistical perturbation analysis^32^ analogous to the construction of individual molecular connectomes. The AHBA dataset was released by the Allen Institute for Brain Science (https://human.brain‐map.org/) and consists of > 20,000 genes’ microarray transcription data across 3702 spatially distinct brain samples taken from six neurotypical adult donors.^37^ Out of these six donors, only two were sampled from both hemispheres and the other four were sampled from only the left hemisphere, considering that no statistically significant hemispheric difference was identified.^37^ Arnatkeviciute et al.^38^ provided a publicly available preprocessed AHBA dataset that includes 10,027 genes’ transcription data from 34 left cortical regions defined by the Desikan–Killiany atlas.^28^ Preprocessing steps taken by Arnatkeviciute et al. mainly include probe‐to‐gene re‐annotation; intensity‐based data filtering; probe selection, accounting for individual variability; and gene filtering.^38^ As shown in Figure S5 in supporting information, we first constructed a reference transcription network $PC_{N}$ by calculating the Pearson correlation across the 10,027 genes for each pair of the 34 left cortical regions. Then, we left the transcriptome data of one gene out to construct a perturbed transcription network $PC_{N-1}$ by re‐calculating the Pearson correlation coefficient across the remaining 10,026 genes. Afterward, we obtained the gene‐specific transcription network of the left‐out gene by calculating the difference between the reference transcription network $PC_{N}$ and the perturbed transcription network $PC_{N-1}$ and normalized it by dividing it by its standard deviation $\frac{1-P{C_{N}}^{2}}{N-1}$.

Then, we trained a gradient tree boosting model to predict the susceptibilities of individual molecular connectomes to AD using these gene‐specific transcription networks as illustrated in Figure S6 in supporting information. For the 561 connections between each pair of the 34 left cortical regions, we randomly divided them into a training dataset including 500 connections and a testing dataset including the remaining 61 connections. The 500 training connections’ strength of the 10,027 gene‐specific transcription networks were fed into a gradient tree boosting system to train separate predictive models of the susceptibility of individual tau and amyloid connectomes. Each gene's contribution to predicting the susceptibilities was determined by the gradient tree boosting model during the training procedure. The optimal predictive models were tested using the 61 testing connections. The accuracy rates of these two predictive models were stably estimated by repeating the randomly selecting training connections and model training and testing procedures 1000 times. We examined the performance of these predictive models by calculating Pearson correlation coefficient between predicted values and true values. The significance levels of these Pearson correlation coefficients were determined using one‐sided sign tests against zero across these 1000 repetitions and were corrected for FDR. A significant correlation between predicted values and true values indicates significant genetic contribution to the susceptibilities of individual molecular connectomes to AD. Each gene's contribution to predicting the susceptibilities was stably estimated by averaging across these 1000 repetitions.

### 3.10 Gene set enrichment analysis

Our connectome‐transcriptome association analysis suggested that the genetic contribution to the susceptibilities of individual molecular connectomes to AD is restricted to genes involved in specific biological processes rather than uniformly distributed across the genome. Thus, we conducted a gene set enrichment analysis to investigate these biological processes in detail.

We first downloaded the human pathway gene sets dataset on November 28, 2023 from http://download.baderlab.org/EM_Genesets/November_07_2023/Human/entrezgene/Human_GO_AllPathways_no_GO_iea_November_07_2023_entrezgene.gmt. The gene sets dataset was collected and updated at least once per month by Reimand et al.^39^ from a set of large, open access and conveniently accessible pathway databases. For each of the 19,524 gene sets in the dataset, we counted the overlapped genes between it and the full list of the 10,027 genes from AHBA based on Entrez Gene ID. We excluded 13,129 gene sets with the count of overlapped genes < 10 or > 200 as suggested by Reimand et al.^39^ and included the remaining 6395 gene sets in the following analysis.

Next, for each of these 6395 gene sets we computed a fold enrichment score by dividing its percentage of contribution to predicting the susceptibilities of individual molecular connectomes to AD by its percentage of the 10,027 genes. Each gene set's percentage of contribution was estimated by summing up the percentage of contribution across genes in it. A gene set with a fold enrichment score > 2 is considered enriched for predicting the susceptibilities. A gene set with a fold enrichment score of < 0.5 is considered unrelated to predicting the susceptibilities.

### 3.11 Gradient tree boosting using XGBoost

In this study, we trained gradient tree boosting models using XGBoost, a scalable tree boosting system with state‐of‐the‐art resource efficiency and superior performance in many machine learning challenges.^40^ Its effectiveness and robustness in brain connectome research have been demonstrated in a previous study.^41^ The implementation of the XGBoost algorithm includes the cross‐validation, training, and testing procedures as shown in Figure S7 in supporting information. The cross‐validation procedure is a pre‐training procedure, which uses the training samples to perform a 10‐fold cross‐validation training to determine the number of “optimal iterations.” Then, the number of “optimal iterations” and the training samples are fed into the training procedure to obtain the optimal model and the contribution of each feature to the optimal model. Finally, this optimal model is tested using the testing samples.

**FIGURE 4:**
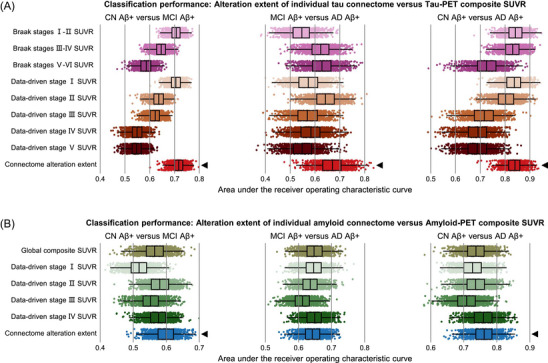
Alterations in individual molecular connectomes outperform previous methods in diagnostic classification. Results from the AUC analysis for binary classification of different diagnostic groups using the alteration extent of individual tau connectome or tau PET composite SUVR (A) and using the alteration extent of individual amyloid connectome or amyloid PET composite SUVR (B). Each dot represents one machine learning model. Box plots depict the interquartile range and the median value of the AUC across 1000 repeated experiments using different training and testing samples. Whiskers extend to the nearest data points ± 1.5 times the interquartile range. We compared the AUC of models using connectome alteration extent to those using composite SUVR value through paired *t* tests. The significance levels of these paired *t* tests were evaluated through 10,000 permutations and corrected for FDR. Black triangles represent significant higher AUC in models using connectome alteration extent than those using composite SUVR values (paired *t* tests, FDR‐corrected *P* values < 0.05). Aβ, amyloid beta; AD, Alzheimer's disease; AUC, area under the receiver operating characteristic curve; CN, cognitively normal; FDR, false discovery rate; MCI, mild cognitive impairment; PET, positron emission tomography; SUVR, standardized uptake value ratio.

**FIGURE 5:**
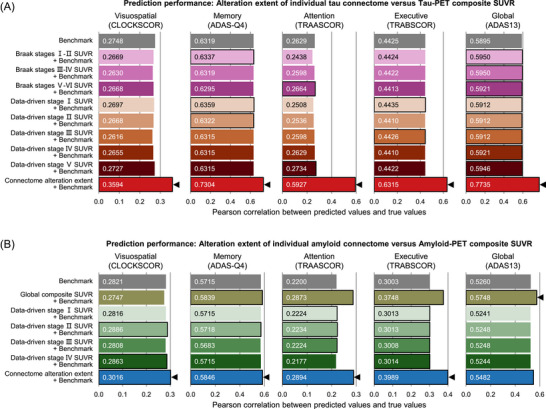
Alterations in individual molecular connectomes outperform previous methods in predicting cognitive decline. Machine learning model's prediction accuracy of longitudinal decline in different cognitive domains and in global cognition before and after adding composite SUVR value or connectome alteration extent of tau (A) and Aβ (B) pathology into the benchmark model. Each bar plot represents a single value. Models that performed better than the benchmark model were highlighted by a black outline. Black triangles represent the best prediction performance. Mean squared and mean absolute errors corresponding to all models are shown in Tables S4 and S5, respectively. Aβ, amyloid beta; PET, positron emission tomography; SUVR, standardized uptake value ratio.

**FIGURE 6:**
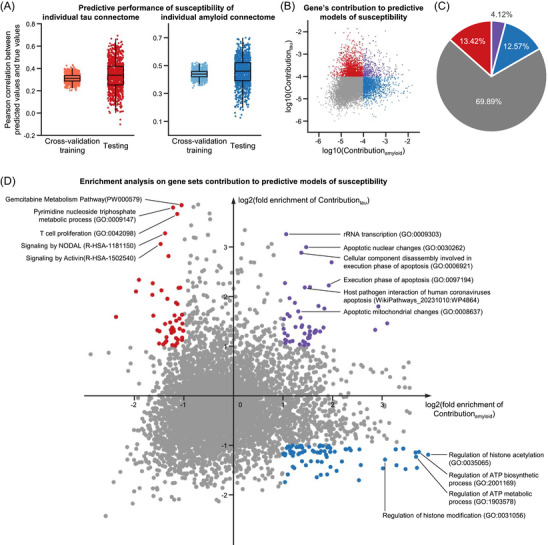
Susceptibilities of individual molecular connectomes to AD are related to specific transcriptomic profiles. A, Machine learning model's prediction accuracy of the susceptibilities of individual tau and amyloid connectomes to AD. Each dot represents one machine learning model. Box plots depict the interquartile range and the median value across 1000 repeated experiments using different training and testing samples. Whiskers extend to the nearest data points ± 1.5 times the interquartile range. B, Gene's contribution to predictive modes of susceptibility. Each dot represents one gene. Different colors index whether the gene contributed higher than the average level to predicting the susceptibility of either individual tau (red) or amyloid (blue) connectome or both (purple). C, Percentage of the four categories of genes shown in (B). D, Fold enrichment score of gene sets contribution to predicting the susceptibilities of individual molecular connectomes to AD. Each dot represents one gene set. Different colors index whether the contribution of the gene set is enriched for predicting the susceptibility of either individual tau (red) or amyloid (blue) connectome or both (purple). $\mathrm{Contributio}n_{\mathrm{tau}}$ and $\mathrm{Contributio}n_{\mathrm{amyloid}}$ in (B) and (D) represent the contribution to predicting the susceptibilities of individual tau and amyloid connectomes to AD, respectively. AD, Alzheimer's disease.

We implemented the XGBoost algorithm using the XGBoost package^40^ v1.7.5.1 in R 4.2.1. For binary classification task of discriminating two diagnostic groups as shown in Figures 2B, C, and 4, we used the following parameters: nrounds = 1500, early_stopping_rounds = 50, nfold = 10, eta = 0.05, objective = “binary:logistic,” eval_metric = “auc.” For multiclass classification task of discriminating three diagnostic groups as shown in Figures S3 and S4, we used the following parameters: nrounds = 1500, early_stopping_rounds = 50, nfold = 10, eta = 0.05, objective = “multi:softprob,” num_class = 3, eval_metric = “auc.” For regression tasks of predicting continuous variables as shown in Figures 5 and 6A, we used the following parameters: nrounds = 1500, early_stopping_rounds = 50, nfold = 10, eta = 0.05, objective = “reg:squarederror.”

### 3.12 Statistical analysis

The effects of diagnostic group on individual molecular connectomes were examined by Kruskal–Wallis tests followed by post hoc one‐sided Wilcoxon rank‐sum tests. The longitudinal effects on individual molecular connectomes were examined by linear mixed‐effects models. The effectiveness of machine learning models was examined by comparing their predictive performance in the 1000 repetitions against the chance level using one‐sided sign tests or by calculating Pearson correlation coefficient between predicted values and true values. Differences in predictive performance between machine learning models were examined by one‐sided paired *t* tests. The significance levels of the Kruskal–Wallis test, rank‐sum tests, and paired *t* tests were evaluated through 10,000 permutations and corrected for FDR. The significance levels of the sign tests, Pearson correlation, and the slope of linear mixed‐effects model were extracted from the MATLAB functions signtest, corr, and fitlme, respectively, and corrected for FDR. We conducted statistical analyses using MATLAB R2023a (https://www.mathworks.com/products/matlab.html), and calculated the individual connectomes using BRAPH software.^42^, ^43^

## 4 RESULTS

### 4.1 Individual molecular connectome mapping using longitudinal PET data

We constructed individual molecular connectomes using longitudinal PET data from ADNI and validated our findings using the HABS cohort. The ADNI cohort included 909 ^18^F‐flortaucipir tau PET scans from 706 individuals and 1521 ^18^F‐florbetapir amyloid PET scans from 1141 individuals (Tables 1 and S1), whereas the HABS cohort included 234 ^18^F‐flortaucipir tau PET scans from 195 individuals and 409 ^11^C‐PiB amyloid PET scans from 308 individuals (Tables 1 and S1). All individuals were diagnosed as being CN or having MCI or AD based on CDR score, cognitive data, and other criteria.^27^, ^31^ As shown in Figure 1A, we used the SUVR of tau pathology in 68 cortical regions defined by the Desikan–Killiany atlas^28^ to construct a reference tau connectome across *N* CN Aβ^−^ subjects. This was conducted by calculating the partial Pearson correlation coefficient between tau PET SUVR values for each pair of cortical regions across these *N* subjects, while controlling for age, sex, and education. Once the reference tau connectome was built, we added an Aβ^+^ subject to the CN Aβ^−^ group and constructed a perturbed tau connectome by re‐calculating the partial Pearson correlation coefficient across these *N*+1 subjects (Figure 1B). Afterward, we obtained the individual tau connectome of this Aβ^+^ subject by calculating the normalized difference between the perturbed tau connectome and the reference tau connectome (Figure 1C). We applied the same procedure to construct the individual amyloid connectome using the amyloid PET SUVR values. We fed these individual tau and amyloid connectomes into the following analysis to examine their validity, sensitivity, and superiority (Figure 1D–H).

### 4.2 Individual molecular connectomes are a unique fingerprint that identifies single subjects

To test the hypothesis that the individual molecular connectome could be used to consistently identify single subjects, we checked whether the connectomes at the initial assessment could identify the same subjects at a later time, and vice versa, through a connectome fingerprinting analysis as illustrated in Figure 1D. We analyzed each Aβ^+^ subject's molecular connectome from the ADNI cohort at the baseline and the first follow‐up. Then, we correlated the connectome of each subject at the baseline with the connectomes of all subjects at the follow‐up, separately for Aβ and tau pathologies. If the connectome at follow‐up showed the highest correlation with the same subject's connectome at baseline, we considered the identification successful (Figure S2). This identification process was performed for each baseline connectome and was repeated in the opposite direction by using each subject's follow‐up connectome to identify the same subject in all baseline connectomes. This analysis showed that we can successfully identify single individuals with high accuracies well above the chance level ($\frac{1}{n}$) within all diagnostic groups (range: 93.44% to 100.00% using individual tau connectomes; range: 94.26% to 100.00% using individual amyloid connectomes; Figure 2A). Independent validation analysis on CN Aβ^+^ subjects from the HABS cohort yielded similar identification accuracies (range: 87.50% to 93.75% using individual tau connectomes; range: 79.03% to 88.71% using individual amyloid connectomes; Figure 2A). Individuals could also be identified with high accuracy when their second follow‐up scan was used instead of their first scan (Figure S8). Thus, similar to a fingerprint, the individual molecular connectome is both unique and reliable for each subject, allowing the identification of individuals, and providing support to the investigation of single subjects using their molecular connectomes.

### 4.3 Individual molecular connectomes discriminate different diagnostic groups

Considering that the individual molecular connectome is both unique and reliable for each subject as shown above, one could speculate that they might enable the discrimination of different diagnostic groups. To test this, we trained machine learning classifiers based on gradient tree boosting to discriminate between Aβ^+^ subjects from different diagnostic groups in ADNI (CN Aβ^+^, MCI Aβ^+^, and AD Aβ^+^) using their molecular connectomes. We observed that these classifiers successfully discriminated different diagnostic groups (area under the receiver operating characteristic curve [AUC] > 0.5, sign tests, FDR‐corrected *P* values < 0.0001; Figure 2B and C). Specifically, these classifiers showed excellent performance in the binary classification of CN Aβ^+^ versus AD Aβ^+^ subjects (individual tau connectome AUC = 0.9112, 95% confidence interval [CI] = 0.8424–0.9644; individual amyloid connectome AUC = 0.8672, 95% CI = 0.8030–0.9190) and moderate performance in the binary classification of CN Aβ^+^ versus MCI Aβ^+^ and MCI Aβ^+^ versus AD Aβ^+^ subjects (AUC = 0.7094–0.7842; Figure 2B and C). These results demonstrate that individual molecular connectomes enable the discrimination of different Aβ^+^ diagnostic groups.

### 4.4 Alterations in individual molecular connectomes increase across the AD continuum and over time

We next examined whether these individual molecular connectomes were altered across the AD continuum as well as longitudinally to assess their value as markers of disease progression. For each Aβ^+^ subject from ADNI, we assessed the alteration extent in the individual molecular connectome using a single value that combines each connection's strength with its contribution to the discrimination of different diagnostic groups (Figures S3 and S4). In this way, a higher alteration extent reflects changes in stronger connections that contributed most to differences between diagnostic groups. We first compared the extent of connectome alteration between different diagnostic groups using the baseline PET scans. This analysis revealed that the alteration extent in the individual molecular connectome was significantly different between different diagnostic groups (Kruskal–Wallis tests, individual tau connectome χ^2^_[359] _= 125.1559, individual amyloid connectome χ^2^_[750] _= 165.1222, FDR‐corrected *P* values < 0.0001; Figure 3A and B), and that the alteration extent was significantly increased across the AD continuum being highest in AD, followed by MCI and CN (post hoc Wilcoxon rank‐sum tests, FDR‐corrected *P* values < 0.0001; Figure 3A and B).

Then, we examined longitudinal connectome alteration using linear mixed‐effects models within each diagnostic group, while controlling for individual‐specific random intercepts and slopes in longitudinal PET scans. We found that the alteration extent in the individual molecular connectome significantly increased over time in each diagnostic group (FDR‐corrected *p* values ≤ 0.0044; Figure 3C and D). Moreover, in the amyloid connectome, we observed a significant time × diagnosis interaction for the AD group (*p* = 0.002) and a trend toward significance for the MCI group (*p* = 0.049), relative to the CN group. This indicates that the longitudinal rate of change was largely attenuated in AD compared to CN (Figure 3D), in line with previous research showing that amyloid accumulation reaches a plateau in advanced AD stages.^44^ When assessing the tau connectome, we found no significant time × diagnosis interaction for either the AD group (*p* = 0.378) or the MCI group (*p* = 0.823), relative to the CN individuals. In contrast to the amyloid connectome, this pattern suggests that tau‐related longitudinal changes are not attenuated and could continue in the later stages of AD.^45^ These findings align with previous evidence^45^ and support the use of tau PET–derived measures to monitor disease progression over time.^46^

Validation analyses demonstrated that the above results are not sensitive to partial volume effects in the tau connectomes (Figure S9 in supporting information). Independent validation analyses on CN Aβ^+^ subjects from the HABS cohort yielded the same alteration trends (Figure S10 in supporting information). Finally, we repeated the analyses with two additional brain atlases^47^, ^48^ and observed similar results (Figures S11 and S12 in supporting information), demonstrating that our findings are robust with respect to the choice of brain parcellation. Altogether, these findings indicate that alterations in individual molecular connectomes significantly increase both across the AD continuum and over time in a consistent manner for different data‐processing strategies and different cohorts.

### 4.5 Alterations in individual molecular connectomes discriminate diagnostic groups better than previous methods

Given the above results, we speculated that alterations in individual molecular connectomes could provide comparable or even higher discriminative power of different diagnostic groups (CN Aβ^+^, MCI Aβ^+^, and AD Aβ^+^) than previous methods. To examine this, we compared the predictive performance of alterations in individual molecular connectomes (single values for each subject combining connection strength with contribution to group classification) to the SUVR values grouped by four sets of composite regions defined by previous studies: three tau PET composite regions for Braak stages (stages I–II, stages III–IV, and stages V–VI),^8^ five tau PET composite regions for data‐driven stages,^9^ a global amyloid PET composite region,^3^ and four amyloid PET composite regions for data‐driven stages.^10^ We observed that models using the alteration extent of individual tau connectome performed significantly better than those using tau PET composite SUVR values in the classification of each pair of diagnostic groups (paired *t* tests on AUC, FDR‐corrected *p* values < 0.0291; Figure 4A). Regarding the individual amyloid connectome, models using the connectome alteration extent performed significantly better than those using amyloid PET composite SUVR values in the classification of CN Aβ^+^ versus MCI Aβ^+^ subjects and CN Aβ^+^ versus AD Aβ^+^ subjects (paired *t* tests on AUC, FDR‐corrected *p* values < 0.0180) but not in the classification of MCI Aβ^+^ versus AD Aβ^+^ subjects (paired *t* tests on AUC, FDR‐corrected P values > 0.05; Figure 4B). These results confirmed that alterations in individual molecular connectomes can provide higher discriminative power of different diagnostic groups than both traditional and data‐driven PET measures in almost all cases.

### 4.6 Alterations in individual molecular connectomes predict better longitudinal cognitive decline than previous methods

We further examined whether alterations in individual molecular connectomes could predict longitudinal cognitive decline to a greater extent than composite SUVR values. We first trained benchmark models based on gradient tree boosting to predict baseline cognitive performance in Aβ^+^ subjects from ADNI using only age, sex, education, and dementia status. These benchmark models performed well during the testing procedure using follow‐up data for all specific cognitive domains and global cognition, indicated by a moderate Pearson correlation coefficient between predicted values and true values (Pearson *R*: 0.2200–0.6319; Figure 5A, B and Tables S4, S5 in supporting information). Then, we added the alteration extent of individual molecular connectomes or the composite SUVR values into these benchmark models. We observed that adding the alteration extent of individual molecular connectomes significantly improved the model performance in all specific cognitive domains and in global cognition (paired *t* tests, FDR‐corrected *p* values < 0.0001; Figure 5A, B and Tables S4, S5), whereas adding composite SUVR values slightly deteriorated the model performance in at least one cognitive domain or in global cognition (Figure 5A, B and Tables S4, S5). This implies that these composite SUVR values cannot provide additional predictive power beyond the combination of age, sex, education, and dementia status in a consistent manner. Together, our findings indicate that alterations in individual molecular connectomes provide higher predictive power of longitudinal cognitive decline than previous methods.

### 4.7 Susceptibilities of individual molecular connectomes to AD are related to specific transcriptomic profiles

According to current global estimates, > 50% of risk to develop AD can be explained by genetic factors.^49^ This strong genetic component implies that the susceptibilities of individual molecular connectomes to AD might be related to specific genetic factors. To investigate this, we performed a connectome–transcriptome association analysis using the AHBA dataset.^37^ First, we estimated the susceptibility of individual tau and amyloid connectomes to AD based on their contributions to the discrimination of different diagnostic groups, respectively (Figures S3 and S4). Next, we developed a new method analogous to the statistical perturbation connectome analysis. We started by constructing a gene‐specific transcription network for each of the 10,027 genes from the preprocessed AHBA dataset^38^ (Figure S5). Then, we trained machine learning predictive models based on gradient tree boosting to predict the susceptibilities of individual tau and amyloid connectomes to AD using the connection strength of these gene‐specific transcription networks (Figures S6 and S7).

We found that the model accurately predicted the susceptibility patterns of individual molecular connectomes to AD by showing significant correlations between the predicted and true values during both the cross‐validation training and testing procedures (sign tests, FDR‐corrected *p* values < 0.0001; Figure 6A). This indicates these predictive models were effective and confirms the genetic contribution to the susceptibilities of individual molecular connectomes to AD. We noted that some genes contributed one or two orders of magnitude more than the average level ($\frac{1}{10,027}$) to these predictive models (Figure 6B). We therefore grouped these genes into three categories according to whether they contributed higher than the average level to predicting the susceptibility of either individual tau (red dots) or amyloid (blue dots) connectome or both (purple dots; Figure 6B). We observed that each of these three categories of genes occupies < 14% of the whole gene list (Figure 6C). These occupancy percentages are lower than the chance level (25%), implying that the genetic contribution to the susceptibilities of individual molecular connectomes to AD is restricted to genes involved in specific biological processes rather than uniformly distributed across the genome. To investigate this in more detail, we conducted a gene set enrichment analysis.

For each of the human pathway gene sets,^39^ we computed a fold enrichment score by dividing its percentage of contribution to predicting the susceptibilities of individual molecular connectomes by its percentage of the 10,027 genes. We observed some gene sets with fold enrichment score greater than two (Figure 6D), confirming that the genetic contribution to the susceptibilities of individual molecular connectomes is restricted to specific biological processes. We also grouped these gene sets into three categories according to whether their contribution is enriched for predicting the susceptibility of either individual tau (red dots) or amyloid (blue dots) connectome or both (purple dots; Figure 6D). We found that gene sets most enriched for predicting the susceptibility of both individual tau and amyloid connectomes to AD are mainly related to apoptosis pathways (Figure 6D), while gene sets most specifically enriched for predicting the susceptibility of either individual tau or amyloid connectome are related to pyrimidine metabolism and histone acetylation, respectively (Figure 6D).

## 5 DISCUSSION

AD is marked by significant inter‐individual variability in the patterns and progression rates of Aβ and tau pathology.^15^ However, most methods applied in AD research and clinical trials use aggregate Aβ and tau measures averaged across brain regions from predefined or data‐driven staging models.^5^, ^6^, ^7^, ^8^, ^9^, ^10^, ^11^, ^12^, ^13^, ^14^ Despite being useful, these models neglect inter‐individual variability and may limit our understanding of AD etiology and progression, hindering the development of personalized therapeutic approaches. In this study, we introduce an individual‐level framework that uses molecular connectomes to characterize the progression of Aβ and tau pathology across the AD continuum. We show that these connectomes are a stable and unique fingerprint for each individual, regardless of the clinical diagnosis. Furthermore, we define a new measure of connectome alteration that shows high discriminative ability both at baseline and in longitudinal analyses, and correlates with cognitive decline over time more strongly than conventional aggregate SUVR measures commonly used in clinical trials. Through the joint longitudinal assessment of Aβ and tau connectomes, and the inclusion of both positive and negative connections, our approach provides a more comprehensive evaluation of AD‐related network alterations compared to previous studies. To investigate the genetic factors shaping these alterations, we developed a method for constructing gene‐specific transcription networks for the brain for all genes in the AHBA dataset. This method enabled connectome–transcriptome analyses that revealed strong associations between molecular connectomes and AD‐relevant transcriptomics profiles, such as apoptosis, pyrimidine metabolism, and histone acetylation. Altogether, our findings demonstrate that individual molecular connectomes accurately monitor disease progression across the AD continuum to a greater extent than other new and well‐established methods, which offers a promising avenue for personalized medicine strategies and testing the effectiveness of current clinical trials in AD.

AD is well known to affect large‐scale brain networks,^50^, ^51^, ^52^ and evidence from functional and structural connectomes has shown that these networks can predict pathological progression over time.^23^, ^53^ However, investigating Aβ and tau molecular connectomes has been challenging due to the static nature of most PET scans, which are typically unsuitable for capturing individual connectomes. To address this, we constructed individual molecular connectomes through a statistical perturbation analysis of single static patient samples against a group of given reference samples. These connectomes capture individual characteristics that are specific to each subject, allowing us to identify single patients. Specifically, we found that it is possible to identify > 83%, 88%, and 98% of CN, MCI, and AD Aβ^+^ individuals, respectively, from a group of subjects using their individual molecular connectomes. Moreover, these individual molecular connectomes showed good performance in the classification of diagnostic groups and detected significant alterations over time. These findings open new possibilities for using PET imaging to understand and track AD at a personalized level.

The use of composite brain regions in previous studies, such as the global Aβ model^3^ or the Braak tau staging model,^8^ has had an important impact in AD, which can now be classified using the A/T scheme by thresholding these Aβ and tau composite values into A+/A– and T+/T–.^46^ Most clinical trials aimed at reducing Aβ and tau pathology used amyloid PET and tau PET SUVR values within these composite regions to determine a patient's Aβ and tau status before and after treatment.^54^, ^55^ However, in our study we observed that these composite SUVR values cannot provide additional predictive power of longitudinal cognitive decline beyond the combination of age, sex, education, and dementia status in a consistent manner, performing well for a few cognitive domains but not for others. Moreover, our findings demonstrated that machine learning models using alterations in individual molecular connectomes outperformed those using composite SUVR values both in discriminating different diagnostic groups and in detecting longitudinal cognitive decline. Similar results were found when we used more recently developed data‐driven staging models.^9^, ^10^ These results highlight the important clinical value of individual molecular connectomes in the detection of Aβ^+^ subjects and their clinical progression. In light of these observations, the plausibility of spatially predefined SUVR composite regions needs to be reconsidered, while personalized approaches deserve further attention in future research and clinical practice.

Connectome–transcriptome association analysis has proven to be a powerful tool for bridging the gap between transcriptional activity of the brain and large‐scale connectome organization in health and disease.^56^ However, almost all previous connectivity studies focused on the co‐transcription network across a large group of genes, such as the pioneering work of Richiardi et al.^57^ The methodology for transcription network analysis at single gene level has not been established to date. By introducing a novel gene‐specific transcription network, here we developed such methodology and evaluated the connection‐wise association between individual molecular connectomes and each gene's transcription network. We found that the susceptibilities of both individual tau and amyloid connectomes to AD are tightly related to a common transcriptomic profile of apoptosis pathways. This is in agreement with the widespread neuronal death due to apoptosis in the brains of patients suffering from AD.^58^ Apoptosis is a predetermined and programmed cellular process, involving an elaborate balance of proapoptotic and antiapoptotic factors.^58^ Both the extracellular Aβ plaques and intracellular hyperphosphorylated tau tangles can destroy the balance and induce an aberrant apoptotic cascade in susceptible brain regions, ending with neuronal loss and neurodegeneration,^59^ which are the primary events occurring during AD progression.^1^

In addition, we observed that the susceptibilities of individual tau and amyloid connectomes to AD are also related to pyrimidine metabolism and histone acetylation, respectively. This agrees well with previous studies on AD showing dysregulation of both pyrimidine metabolism^60^ and histone acetylation.^61^ Pyrimidine metabolism provides a broad spectrum of key molecules that participate in diverse cellular functions, such as the synthesis and repair of DNA and lipids.^62^ The disruption in pyrimidine metabolism pathways might be responsible for DNA damage, especially the mitochondrial DNA damage observed in AD brains.^63^ Specifically, it has been found that mitochondrial dysfunction pushes amyloid precursor protein processing toward Aβ production and affects tau phosphorylation,^64^ which is believed to be achieved through a decrease in the *de novo* synthesis of pyrimidine nucleotides.^65^ Histone acetylation and deacetylation are covalent enzymes that activate and repress gene transcription, respectively, whose dysregulation is involved in a variety of signal transduction pathways targeted by AD progression, such as those responsible for cell apoptosis, neuronal plasticity, and inflammation.^66^ Therefore, histone acetylation is believed to play a neuroprotective role by mediating Aβ‐induced apoptotic neuronal reaction in the central nervous system,^67^ which supports it as one of the epigenetic modifications or markers in the etiology of AD.^66^ Drugs that can modify histone acetylation, such as histone deacetylase inhibitors, are currently being explored for their potential to restore healthy gene expression patterns and ameliorate symptoms or slow the progression of AD.^68^ Accordingly, these findings indicate that the susceptibilities of individual molecular connectomes correlate closely with transcriptomic profiles that are directly relevant to AD.

Some results of the present study should be interpreted with caution. First, we conducted a data‐driven analysis of connectome–transcriptome associations using the AHBA dataset. For the ≈ 20,000 available genes from the AHBA dataset, more than half were excluded in data preprocessing,^38^ which may have induced incomplete observations in our data‐driven analysis. Second, our transcriptomic analysis only addressed the association between individual molecular connectomes and transcriptomic profiles but did not explore causal relationships between them. Hence, the potential causalities underlying this association should be clarified before translating our findings into the clinical setting, which could be achieved in animal models of AD.^69^ Third, we developed a connection‐wise methodology in the connectome–transcriptome association analysis rather than adopting a region‐wise framework as in previous studies.^70^, ^71^ This newly developed methodology captured evidence observed in previous AD pathophysiological studies.^58^, ^59^, ^60^, ^61^, ^63^, ^64^, ^65^, ^66^, ^67^, ^68^ Furthermore, we did not assess whether there are any partial volume effects in the amyloid connectome as ADNI does not currently provide any partial volume correction (PVC) on the amyloid PET data. Nevertheless, there is currently no consensus on whether PVC is necessary for amyloid PET analyses,^72^ with some studies reporting differences, while others have found no significant differences in their findings after applying PVC.^72^, ^73^ In fact, previous studies have pointed out that special caution is needed when applying PVC to amyloid PET images, as different correction methods could introduce different biases that could lead to erroneous estimations about regional uptakes.^74^, ^75^ Finally, the cohorts included in our study consist of individuals across the AD symptomatic spectrum (CN, MCI, and AD) that were designed to capture different stages of the more common, sporadic, and late‐onset form of the disease. Future studies examining early onset AD cases with our approach should be performed to assess the sensitivity of our method across the complete syndromic heterogeneity of AD.^1^

## AUTHOR CONTRIBUTIONS

*Conceptualization*: Z.X., J.B.P. *Methodology*: Z.X., J.B.P., M.M., Y.W.C., M.V., A.S., G.V., S.G.P. *Investigation*: Z.X. *Visualization*: Z.X., J.W.S. *Funding acquisition*: J.B.P., S.G.P. *Supervision*: J.B.P. *Writing—original draft*: Z.X., J.B.P. *Writing—review & editing*: J.B.P., Z.X., M.M., J.W.S., G.V., M.V., A.S., Y.W.C., S.G.P., M.S.

## CONFLICT OF INTEREST STATEMENT

The authors declare no conflicts of interest. Author disclosures are available in the supporting information.

## CONSENT STATEMENT

Informed written consent was obtained from all participants in ADNI and HABS by each cohort's investigators. All protocols were approved by each cohort's respective institutional ethical review board.

## Supporting information



Supporting Information

Supporting Information

## Data Availability

The ADNI cohort is available through the ADNI data portal (https://adni.loni.usc.edu/), subject to approval by the ADNI team. The HABS cohort is available through the XNAT data portal (https://central.xnat.org/), subject to approval by the HABS team. The preprocessed AHBA dataset is available at https://doi.org/10.6084/m9.figshare.6852911. The human pathway gene sets dataset is available at http://download.baderlab.org/EM_Genesets/. The code to reproduce the results reported in this article is available at https://github.com/zhileixu/IndividualMolecularConnectomes.
