# Supplementary material for: Mapping individual molecular connectomes in Alzheimer's disease
Source: Alzheimers Dement. 2026 Mar 26;22(3):e71310. doi: 10.1002/alz.71310 (PMC13093537; doi:10.1002/alz.71310)
Supplement: Supplementary file 2 — Supporting Information [file ALZ-22-e71310-s002.docx]

Supplementary Materials for

**Mapping Individual Molecular Connectomes in Alzheimer’s Disease**

Zhilei Xu *et al.*

*Corresponding authors: Zhilei Xu, Email: zhilei.xu@ki.se // Joana B. Pereira, Email: joana.pereira@ki.se

This file includes:

Tables S1 to S5

Figs. S1 to S12

**Table S1. ﻿Group-wise statistical comparisons of demographic and clinical characteristics of the included participants.**

|  | **Participants with tau-PET scans** | | | | **Participants with amyloid-PET scans** | | | |
| --- | --- | --- | --- | --- | --- | --- | --- | --- |
|  | **CN Aβ-** | **CN Aβ+** | **MCI Aβ+** | **AD Aβ+** | **CN Aβ-** | **CN Aβ+** | **MCI Aβ+** | **AD Aβ+** |
| **ADNI cohort** | | | | | | | | |
| N | 344 | 133 | 142 | 87 | 388 | 177 | 324 | 252 |
| Scans | 344 | 236 | 204 | 125 | 388 | 313 | 502 | 318 |
| Age at first scan | - | <0.001 | <0.001 | <0.001 | - | <0.001 | <0.001 | <0.001 |
| Sex | - | 0.179 | 0.116 | 0.012 | - | 0.051 | 0.022 | 0.045 |
| Education | - | 0.378 | 0.015 | <0.001 | - | 0.474 | 0.021 | <0.001 |
| MMSE at first scan | - | 0.499 | <0.001 | <0.001 | - | 0.245 | <0.001 | <0.001 |
| **HABS cohort** | | | | | | | | |
| N | 132 | 63 | - | - | 207 | 101 | - | - |
| Scans | 132 | 102 | - | - | 207 | 202 | - | - |
| Age at first scan | - | 0.018 | - | - | - | <0.001 | - | - |
| Sex | - | 0.870 | - | - | - | 0.619 | - | - |
| Education | - | 0.504 | - | - | - | 0.252 | - | - |
| MMSE at first scan | - | 0.027 | - | - | - | 0.001 | - | - |

P-values represent statistical comparisons of each Aβ+ group against the CN Aβ- reference group within the ADNI and HABS cohorts. Mann–Whitney U tests were used for continuous variables and chi-square tests for categorical variables. MMSE, Mini Mental State Examination. P-values correspond to values shown in Table 1 in the main manuscript.

**Table S2. Demographic and clinical characteristics of the included participants with tau-PET scans after partial volume correction from ADNI.**

|  | **CN Aβ-** | **CN Aβ+** | **MCI Aβ+** | **AD Aβ+** | **All** |
| --- | --- | --- | --- | --- | --- |
| N | 344 | 135 | 143 | 88 | 710 |
| Scans | 344 | 239 | 205 | 127 | 915 |
| Age at first scan (years) | 70.8 (50.5-94.2) | 73.9 (56.5-91.4) | 75.5 (56.0-92.4) | 76.9 (55.5-93.9) | 73.2 (50.5-94.2) |
| Follow-up scans | - | 1 (1-4) | 1 (1-3) | 1 (1-2) | 1 (1-4) |
| Follow-up years | - | 2.1 (0.8-5.4) | 2.1 (0.6-4.9) | 1.3 (0.5-3.0) | 2.0 (0.5-5.4) |
| Sex (male/female) | 150/194 | 50/85 | 73/70 | 51/37 | 324/386 |
| Education (years) | 17.0 (11.0-20.0) | 16.0 (12.0-20.0) | 16.0 (12.0-20.0) | 16.0 (10.0-20.0) | 16.0 (10.0-20.0) |
| MMSE at first scan | 29.0 (23.0-30.0) | 29.0 (24.0-30.0) | 27.0 (19.0-30.0) | 23.0 (9.0-30.0) | 29.0 (9.0-30.0) |

Data presented in the table are reported as median (range), unless otherwise stated.

**Table S3. Composite brain region label provided by previous studies.**

| **Brain region** | **Braak stage label** | **Data driven tau stage label** | **^18^F-florbetapir global composite region label** | **Data driven amyloid stage label** |
| --- | --- | --- | --- | --- |
| superiorfrontal | Ⅴ | Ⅳ | 1 | Ⅱ |
| rostralmiddlefrontal | Ⅴ | Ⅳ | 1 | Ⅱ |
| caudalmiddlefrontal | Ⅴ | Ⅲ | 1 | Ⅱ |
| parsopercularis | Ⅴ | Ⅳ | 1 | Ⅲ |
| parstriangularis | Ⅴ | Ⅳ | 1 | Ⅱ |
| parsorbitalis | Ⅴ | Ⅳ | 1 | Ⅲ |
| lateralorbitofrontal | Ⅴ | Ⅳ | 1 | Ⅱ |
| medialorbitofrontal | Ⅴ | Ⅳ | 1 | Ⅱ |
| precentral | Ⅵ | Ⅴ | 1 | Ⅲ |
| paracentral | Ⅵ | Ⅴ | 1 | Ⅲ |
| frontalpole | - | Ⅳ | 1 | Ⅱ |
| superiorparietal | Ⅴ | Ⅲ | 1 | Ⅲ |
| inferiorparietal | Ⅴ | Ⅲ | 1 | Ⅲ |
| supramarginal | Ⅴ | Ⅲ | 1 | Ⅱ |
| postcentral | Ⅵ | Ⅴ | 1 | Ⅲ |
| precuneus | Ⅴ | Ⅲ | 1 | Ⅱ |
| superiortemporal | Ⅴ | Ⅱ | 1 | Ⅱ |
| middletemporal | Ⅳ | Ⅱ | 1 | Ⅱ |
| inferiortemporal | Ⅳ | Ⅱ | 1 | Ⅰ |
| bankssts | - | Ⅱ | 1 | Ⅱ |
| fusiform | Ⅲ | Ⅱ | 1 | Ⅰ |
| transversetemporal | - | Ⅴ | 1 | Ⅲ |
| entorhinal | Ⅰ-Ⅱ | Ⅰ | 1 | Ⅲ |
| temporalpole | - | Ⅱ | 1 | Ⅱ |
| parahippocampal | Ⅲ | Ⅰ | 1 | Ⅲ |
| lateraloccipital | Ⅴ | Ⅲ | 1 | Ⅱ |
| lingual | Ⅴ | Ⅴ | 1 | Ⅳ |
| cuneus | Ⅵ | Ⅴ | 1 | Ⅲ |
| pericalcarine | Ⅵ | Ⅴ | 1 | Ⅲ |
| rostralanteriorcingulate | Ⅴ | Ⅲ | 1 | Ⅰ |
| caudalanteriorcingulate | Ⅴ | Ⅳ | 1 | Ⅰ |
| posteriorcingulate | Ⅴ | Ⅳ | 1 | Ⅱ |
| isthmuscingulate | - | Ⅲ | - | Ⅱ |
| insula | Ⅴ | Ⅳ | - | Ⅱ |

**Table S4. ﻿Mean squared error for models shown in Figure 5.**

| **Model** | **Visuospatial** | **Memory** | **Attention** | **Executive** | **Global** |
| --- | --- | --- | --- | --- | --- |
| **Alteration extent of individual tau connectome versus Tau-PET composite SUVR** | | | | | |
| Benchmark | 1.22 | 5.95 | 559.46 | 4701.65 | 96.80 |
| Braak stages Ⅰ-Ⅱ SUVR + Benchmark | 1.23 | 5.93 | 565.32 | 4676.83 | 97.84 |
| Braak stages Ⅲ-Ⅳ SUVR + Benchmark | 1.23 | 5.95 | 559.69 | 4617.73 | 97.84 |
| Braak stages Ⅴ-Ⅵ SUVR + Benchmark | 1.23 | 5.98 | 559.17 | 4639.52 | 97.36 |
| Data-driven stage Ⅰ SUVR + Benchmark | 1.23 | 5.92 | 561.20 | 4713.63 | 97.21 |
| Data-driven stage Ⅱ SUVR + Benchmark | 1.23 | 5.95 | 560.39 | 4649.82 | 97.21 |
| Data-driven stage Ⅲ SUVR + Benchmark | 1.23 | 5.95 | 559.69 | 4689.04 | 97.21 |
| Data-driven stage Ⅳ SUVR + Benchmark | 1.23 | 5.95 | 559.46 | 4649.82 | 97.36 |
| Data-driven stage Ⅴ SUVR + Benchmark | 1.12 | 4.60 | 378.42 | 3455.69 | 62.21 |
| **Alteration extent of individual amyloid connectome versus Amyloid-PET composite SUVR** | | | | | |
| Benchmark | 1.27 | 6.84 | 632.05 | 4980.86 | 126.35 |
| Global composite SUVR + Benchmark | 1.31 | 6.84 | 595.25 | 4610.92 | 117.26 |
| Data-driven stage Ⅰ SUVR + Benchmark | 1.27 | 6.85 | 629.16 | 5007.78 | 126.81 |
| Data-driven stage Ⅱ SUVR + Benchmark | 1.28 | 6.84 | 627.97 | 5007.78 | 126.91 |
| Data-driven stage Ⅲ SUVR + Benchmark | 1.27 | 6.97 | 629.16 | 4998.78 | 126.91 |
| Data-driven stage Ⅳ SUVR + Benchmark | 1.28 | 6.85 | 634.87 | 4989.61 | 127.04 |
| Connectome alteration extent + Benchmark | 1.28 | 6.79 | 604.57 | 4433.65 | 122.55 |

**Table S5. ﻿Mean absolute error for models shown in Figure 5.**

| **Model** | **Visuospatial** | **Memory** | **Attention** | **Executive** | **Global** |
| --- | --- | --- | --- | --- | --- |
| **Alteration extent of individual tau connectome versus Tau-PET composite SUVR** | | | | | |
| Benchmark | 0.84 | 1.89 | 13.68 | 46.67 | 6.38 |
| Braak stages Ⅰ-Ⅱ SUVR + Benchmark | 0.83 | 1.88 | 13.91 | 46.73 | 6.38 |
| Braak stages Ⅲ-Ⅳ SUVR + Benchmark | 0.82 | 1.89 | 13.69 | 46.95 | 6.38 |
| Braak stages Ⅴ-Ⅵ SUVR + Benchmark | 0.82 | 1.89 | 13.66 | 46.89 | 6.38 |
| Data-driven stage Ⅰ SUVR + Benchmark | 0.83 | 1.88 | 13.79 | 46.66 | 6.38 |
| Data-driven stage Ⅱ SUVR + Benchmark | 0.82 | 1.89 | 13.76 | 46.83 | 6.38 |
| Data-driven stage Ⅲ SUVR + Benchmark | 0.81 | 1.89 | 13.69 | 46.72 | 6.38 |
| Data-driven stage Ⅳ SUVR + Benchmark | 0.82 | 1.89 | 13.68 | 46.83 | 6.38 |
| Data-driven stage Ⅴ SUVR + Benchmark | 0.76 | 1.65 | 12.16 | 41.17 | 5.14 |
| **Alteration extent of individual amyloid connectome versus Amyloid-PET composite SUVR** | | | | | |
| Benchmark | 0.86 | 2.15 | 16.01 | 51.30 | 7.97 |
| Global composite SUVR + Benchmark | 0.88 | 2.11 | 15.16 | 48.78 | 7.63 |
| Data-driven stage Ⅰ SUVR + Benchmark | 0.86 | 2.15 | 15.84 | 51.86 | 7.94 |
| Data-driven stage Ⅱ SUVR + Benchmark | 0.88 | 2.15 | 15.74 | 51.86 | 7.90 |
| Data-driven stage Ⅲ SUVR + Benchmark | 0.86 | 2.17 | 15.84 | 51.70 | 7.90 |
| Data-driven stage Ⅳ SUVR + Benchmark | 0.87 | 2.15 | 16.10 | 51.58 | 7.89 |
| Connectome alteration extent + Benchmark | 0.87 | 2.14 | 15.85 | 47.28 | 7.94 |


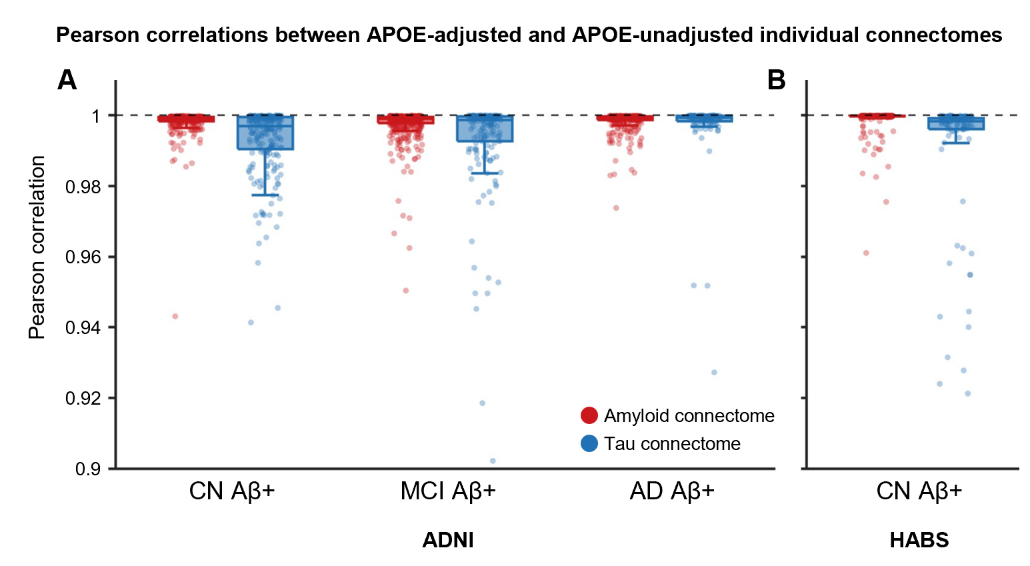


**Figure S1. Effect of APOE on individual molecular connectomes.** Correlation between connectivity strengths of individual connectomes calculated with and without APOE included as a covariate, in addition to age, sex, and education. Each point represents the correlation between the two connectomes for an individual participant, with amyloid connectomes shown in red and tau connectomes in blue, for both (**A**) ADNI and (**B**) HABS cohort.

**
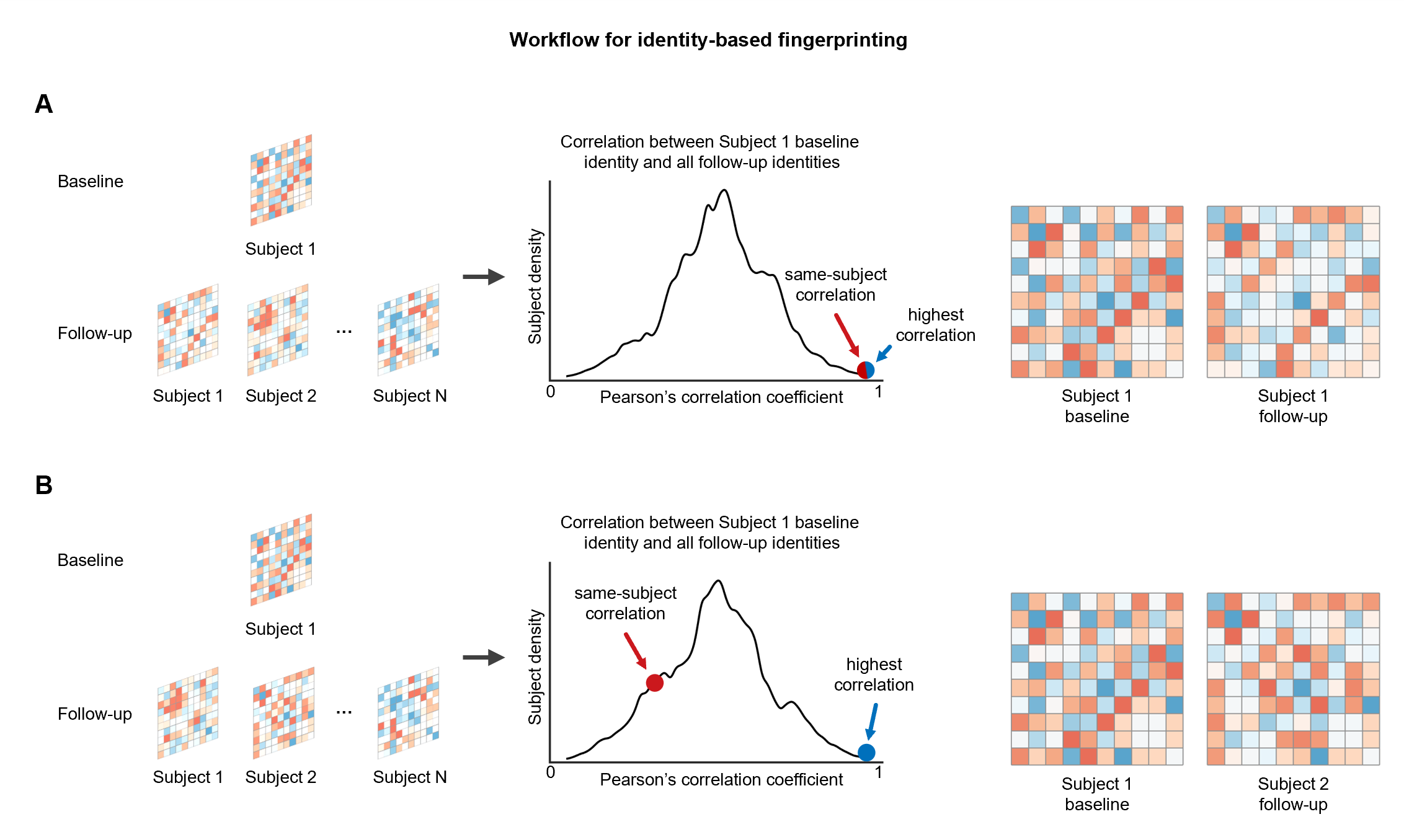
**

**Figure S2. Fingerprinting and individual identity.** An individual’s identity is the set of the unique, pairwise connections in his/her molecular connectivity matrix. (A) Successful fingerprinting. Subject 1’s connectivity at baseline is correlated with the follow-up connectivity matrices of all participants, producing a distribution of Pearson’s correlation coefficient values (black curve). Fingerprinting is considered successful when the correlation between the baseline and follow-up connectivity of Subject 1 (same-subject correlation, red circle) is also the highest correlation observed among all possible matches (blue circle). In this case, the participant’s follow-up identity is most similar to their own baseline identity. (B) Fingerprinting is unsuccessful when at least one other participant shows a higher similarity to Subject 1’s baseline identity than Subject 1’s own follow-up. In this example, Subject 2’s follow-up matrix has a correlation greater than Subject 1’s same-subject correlation, leading to a mis-identification.

**
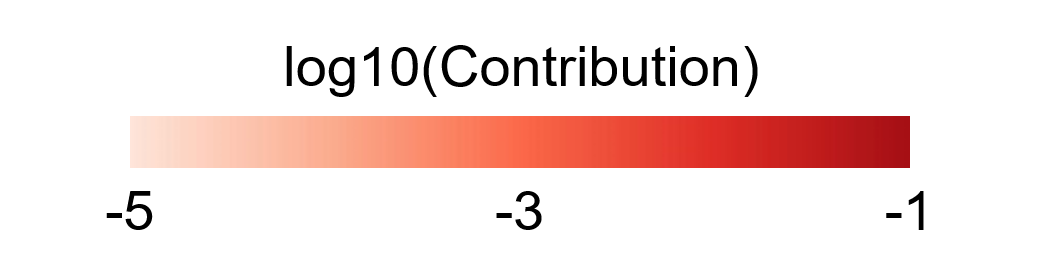
**

**Fig. S3. Connection’s contribution to the discrimination of three diagnostic groups using individual tau connectome.**

**
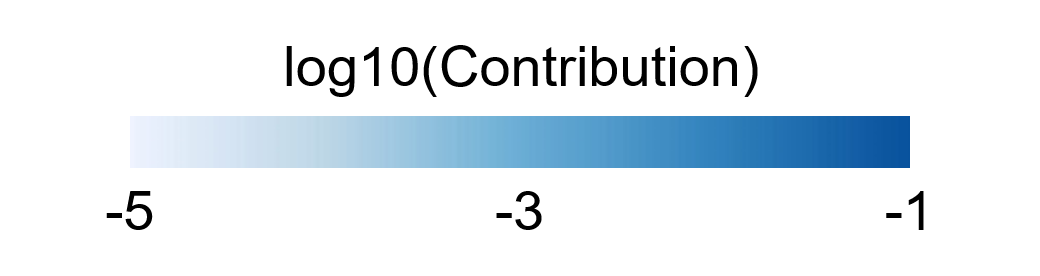
**

**Fig. S4. Connection’s contribution to the discrimination of three diagnostic groups using individual amyloid connectome.**

**Fig. S5. The workflow for constructing gene-specific transcription network.** (**A**), A reference transcription network was first constructed across *N*=10,027 genes by computing the Pearson correlation coefficient $\mathrm{PC}_{N}$ between transcription levels for each pair of cortical regions. (**B**), Then a perturbed transcription network $\mathrm{PC}_{N-1}$ was constructed by leaving one gene out from the above *N* genes and re-calculating the Pearson correlation coefficient across the remaining *N*-1 genes. **c**, The gene-specific transcription network for the left-out gene was obtained through dividing the difference between the reference transcription network $\mathrm{PC}_{N}$ and the perturbed transcription network $\mathrm{PC}_{N-1}$ by its standard deviation $\frac{1-{\mathrm{PC}_{N}}^{2}}{N-1}$.

**
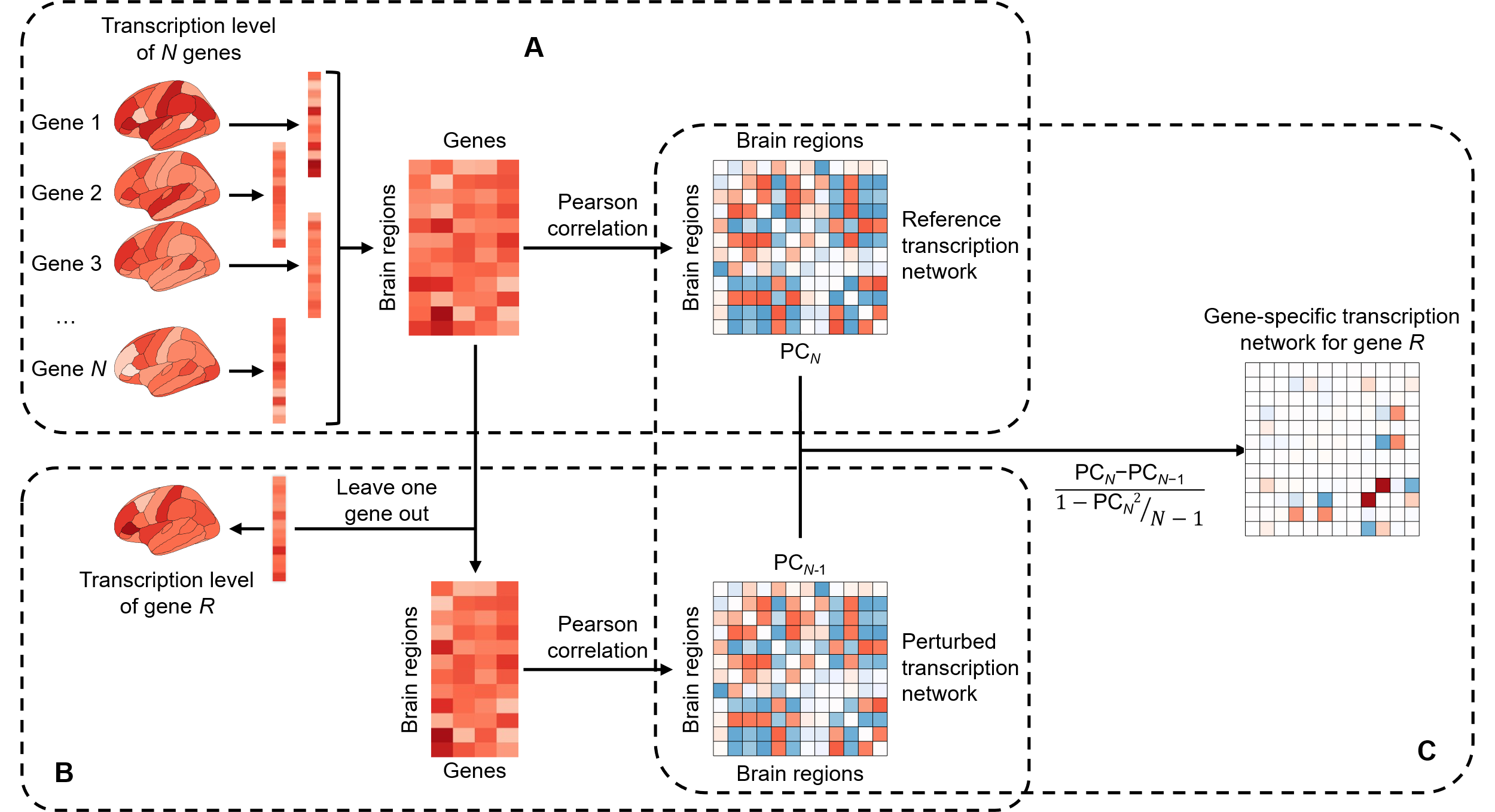
**

**
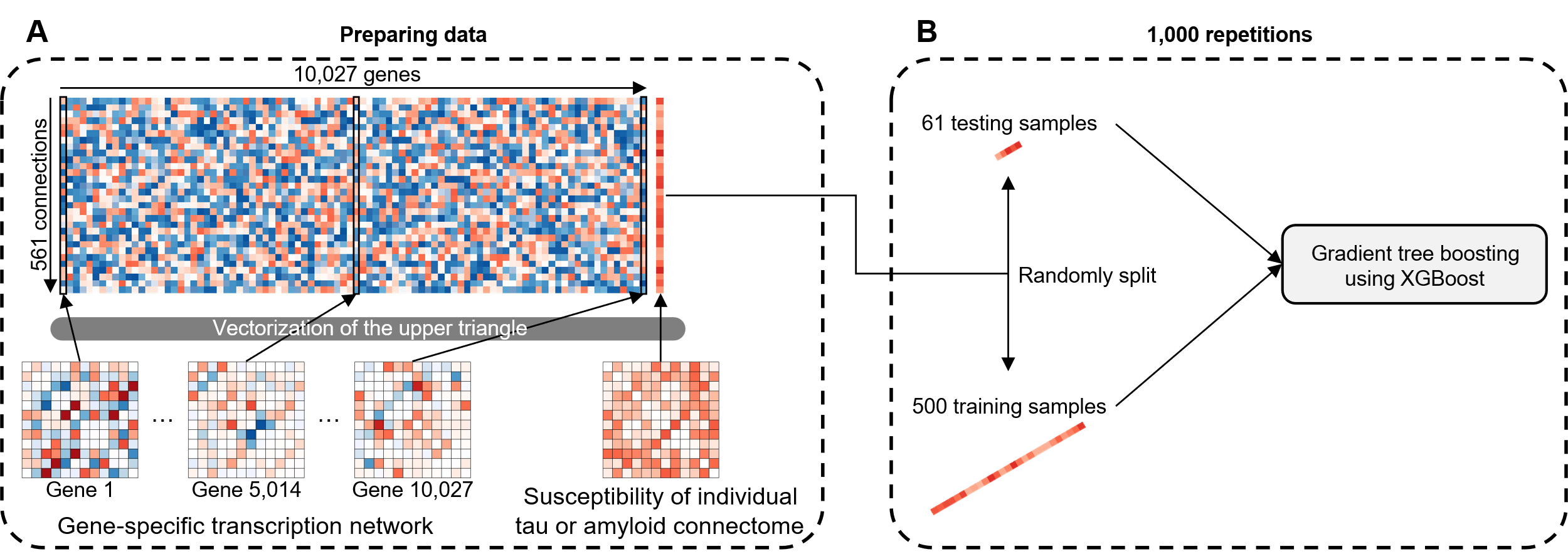
**

**Fig. S6. The workflow for predicting the susceptibilities of individual molecular connectomes to AD using gene-specific transcription networks.** (**A**), The upper triangle of each gene-specific transcription network was first vectorized and was collected into a 561×10,027 matrix that represents the 561 connections’ strength of the 10,027 genes’ transcription network. The upper triangle of the susceptibility of individual tau or amyloid connectome was vectorized and was matched with each of the 561 connections. (**B**), Then these 561 connections were randomly split into training and testing samples that were fed into a gradient tree boosting model to predict their corresponding susceptibilities using the connection strength of the 10,027 genes’ transcription network. The randomly splitting training and testing samples and gradient tree boosting model training and testing procedures were repeated 1,000 times.

**
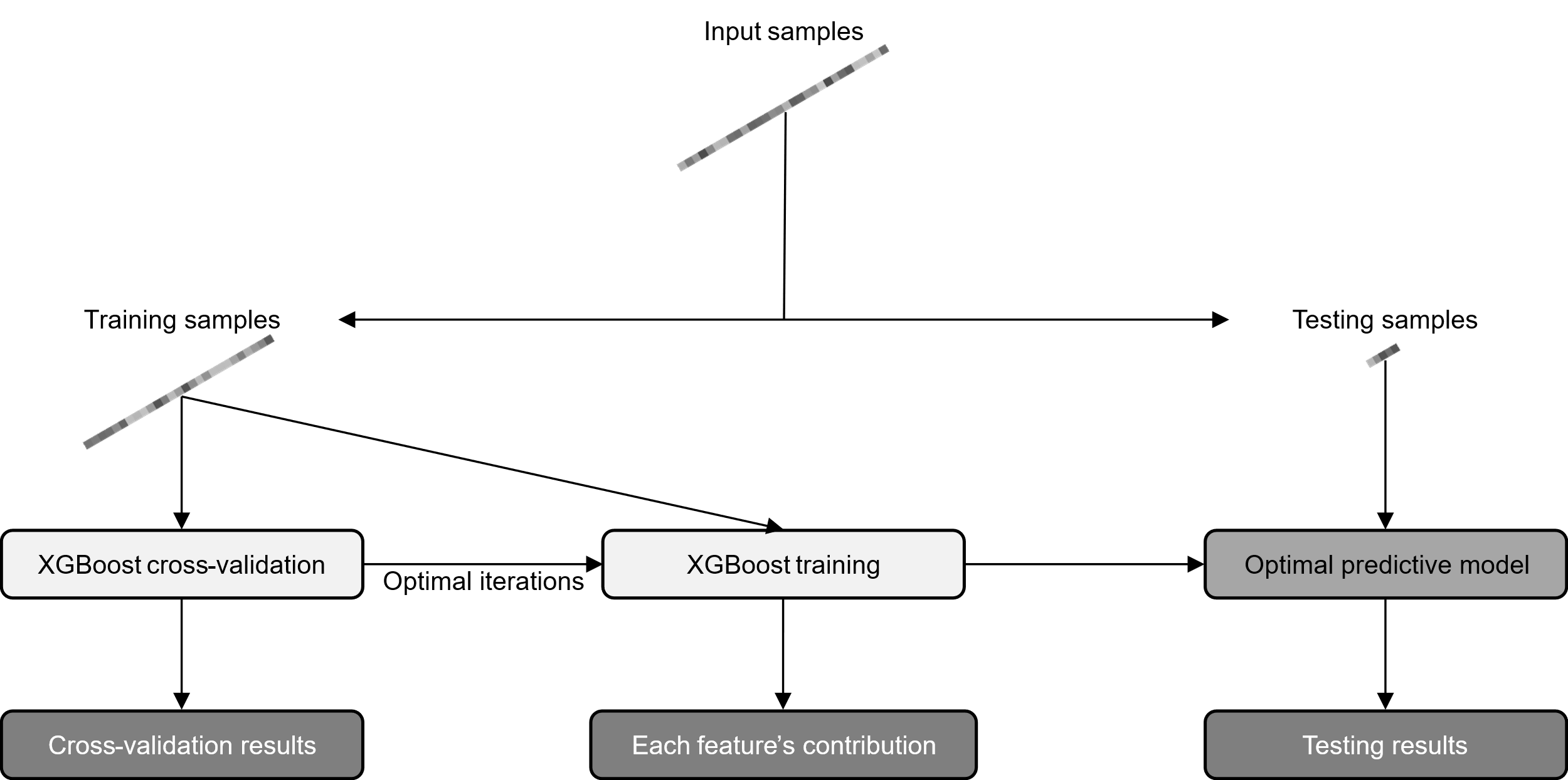
**

**Fig. S7. Gradient tree boosting using XGBoost.** The training samples are first used to perform a 10-fold cross-validation training to determine the number of “optimal iterations”. Then, the number of “optimal iterations” and the training samples are fed into the training procedure to obtain the optimal model and the contribution of each feature to the optimal model. Finally, this optimal model is tested using the testing samples.


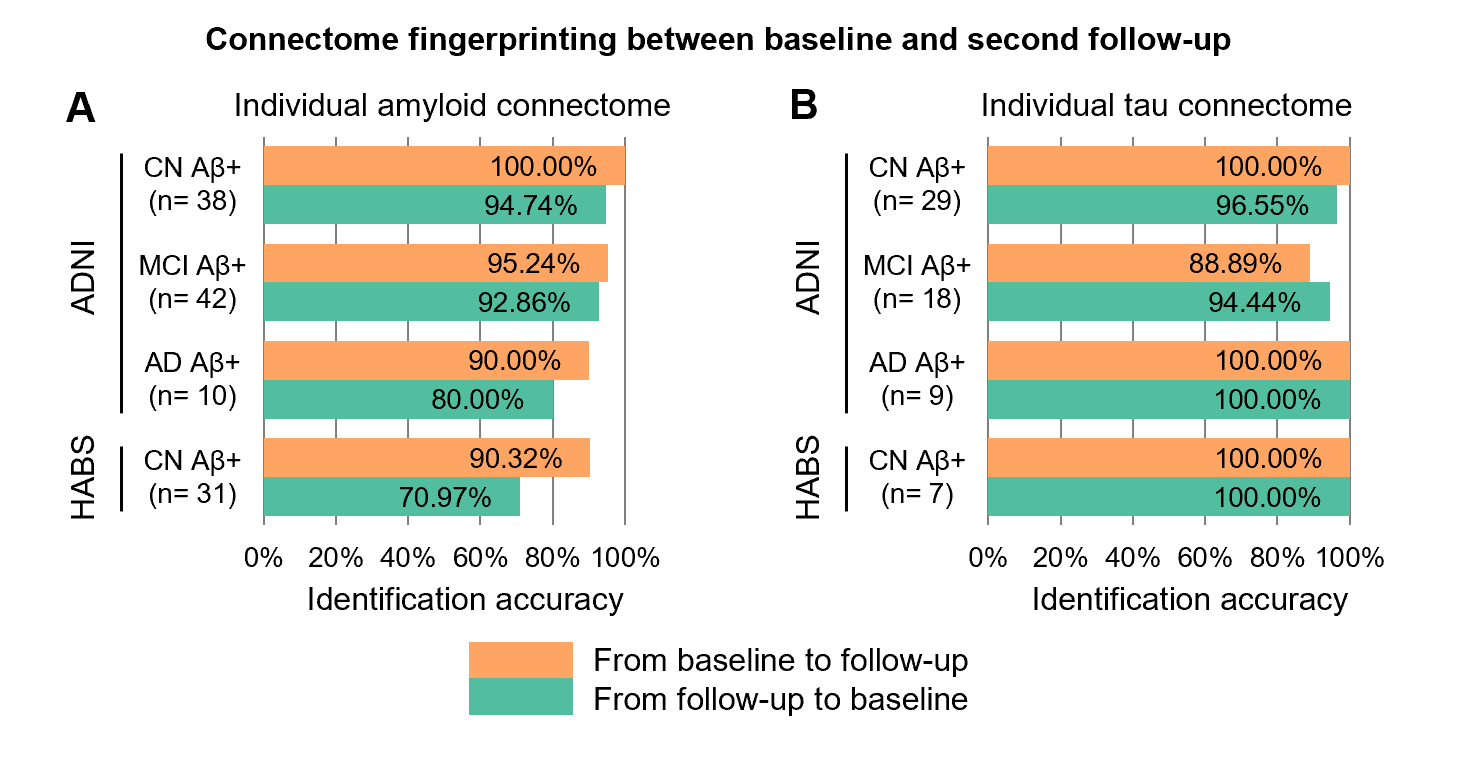


**Figure S8. Connectome fingerprinting using individual molecular connectomes**. Connectome fingerprinting accuracy within each diagnostic group using (**A**) amyloid and (**B**) tau individual connectomes. The fingerprinting analysis was performed between the baseline and the second follow-up scan. Compare with Figure 2A in the main manuscript.

**
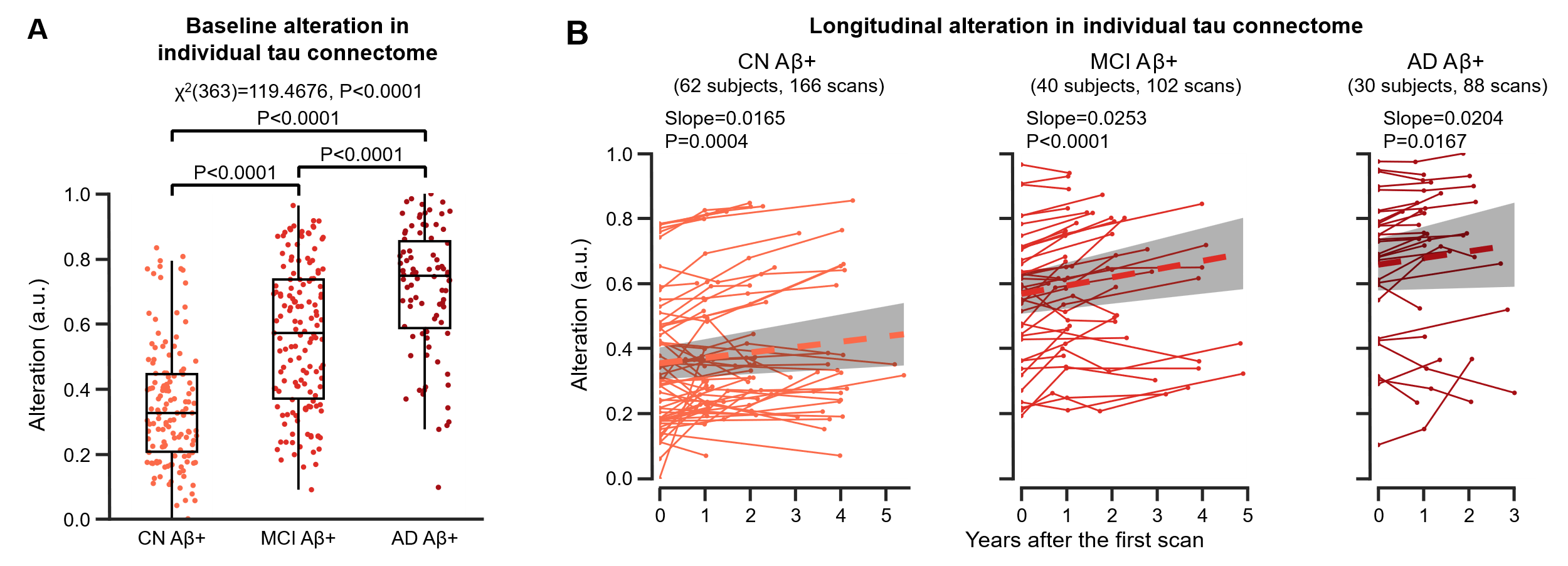
**

**Fig. S9. Alterations in individual tau connectome increase across the AD continuum and over time.** (**A**), The extent of alteration in individual tau connectome across the AD continuum using data after partial volume correction. Each dot represents one subject. Box plots depict the interquartile range and the median value of the distribution. Whiskers extend to the nearest data points ±1.5-times the interquartile range. The effects of diagnostic group (CN Aβ+, MCI Aβ+, and AD Aβ+) on individual tau connectome were examined by Kruskal-Wallis test followed by post hoc Wilcoxon rank-sum tests. The significance levels of the Kruskal-Wallis test and post hoc Wilcoxon rank-sum tests were evaluated through 10,000 permutations and were corrected for FDR. (**B**), Longitudinal alteration in individual tau connectome across the AD continuum using data after partial volume correction. Each dot represents one tau-PET scan. Each thin line connects tau-PET scans from one subject. Bold dashed line and grey shading represent the fitted line and its 95% confidence intervals from the linear mixed-effects model. P values were extracted from the linear mixed-effects model and were corrected for FDR. CN, cognitively normal; MCI, mild cognitive impairment; AD, Alzheimer’s disease; Aβ+, amyloid-β positive; a.u. arbitrary unit.

**
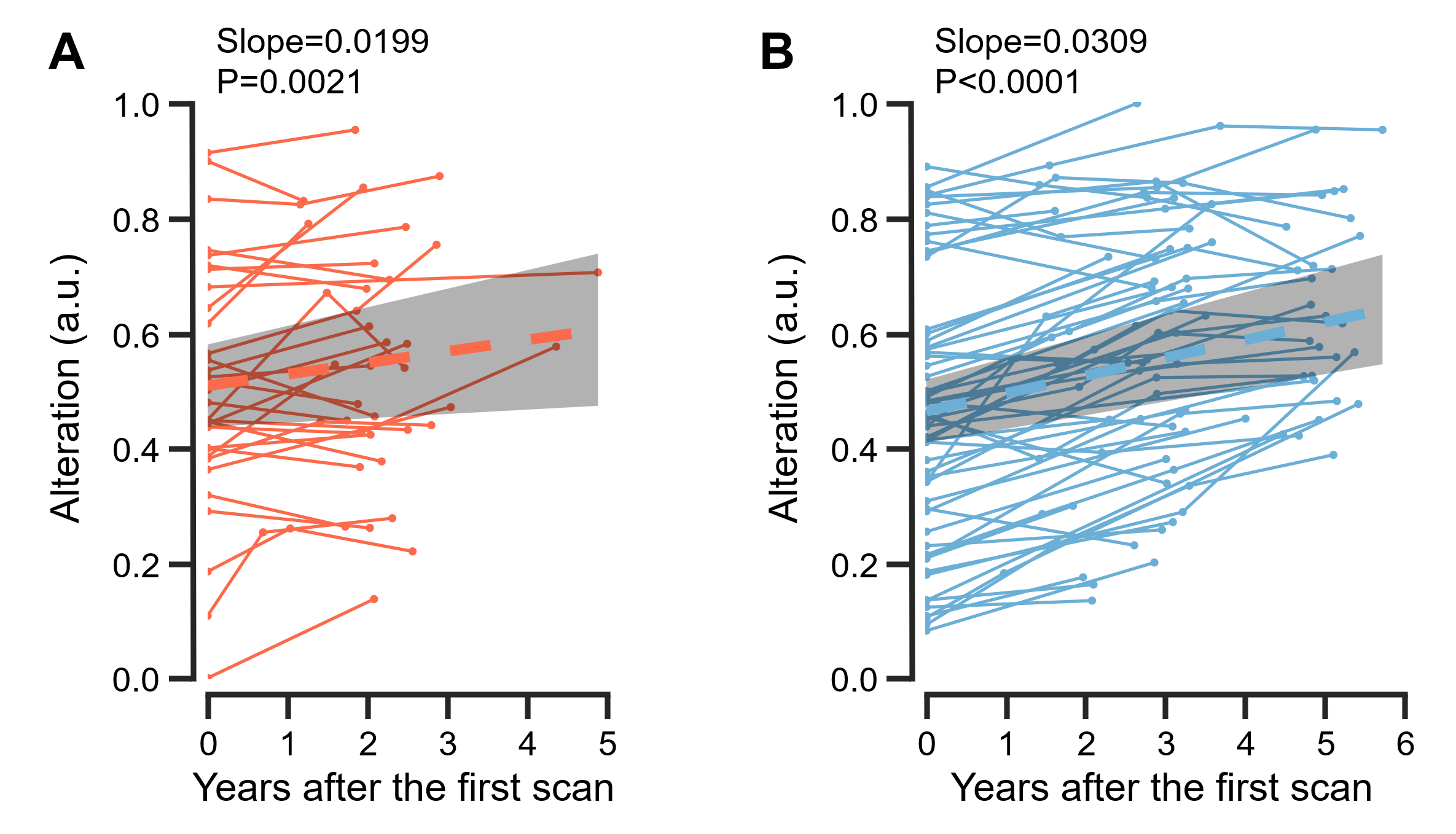
**

**Fig. S10. Alterations in individual molecular connectomes from the HABS cohort increase over time.** Longitudinal alterations in individual tau (**A**) and amyloid (**B**) connectomes for cognitively normal amyloid-β positive subjects from the HABS cohort (71 tau-PET scans from 32 subjects, 163 amyloid-PET scans from 62 subjects). Each dot represents one PET scan. Each thin line connects PET scans from one subject. Bold dashed line and grey shading represent the fitted line and its 95% confidence intervals from the linear mixed-effects model. P values were extracted from the linear mixed-effects model and were corrected for FDR. a.u. arbitrary unit.

**
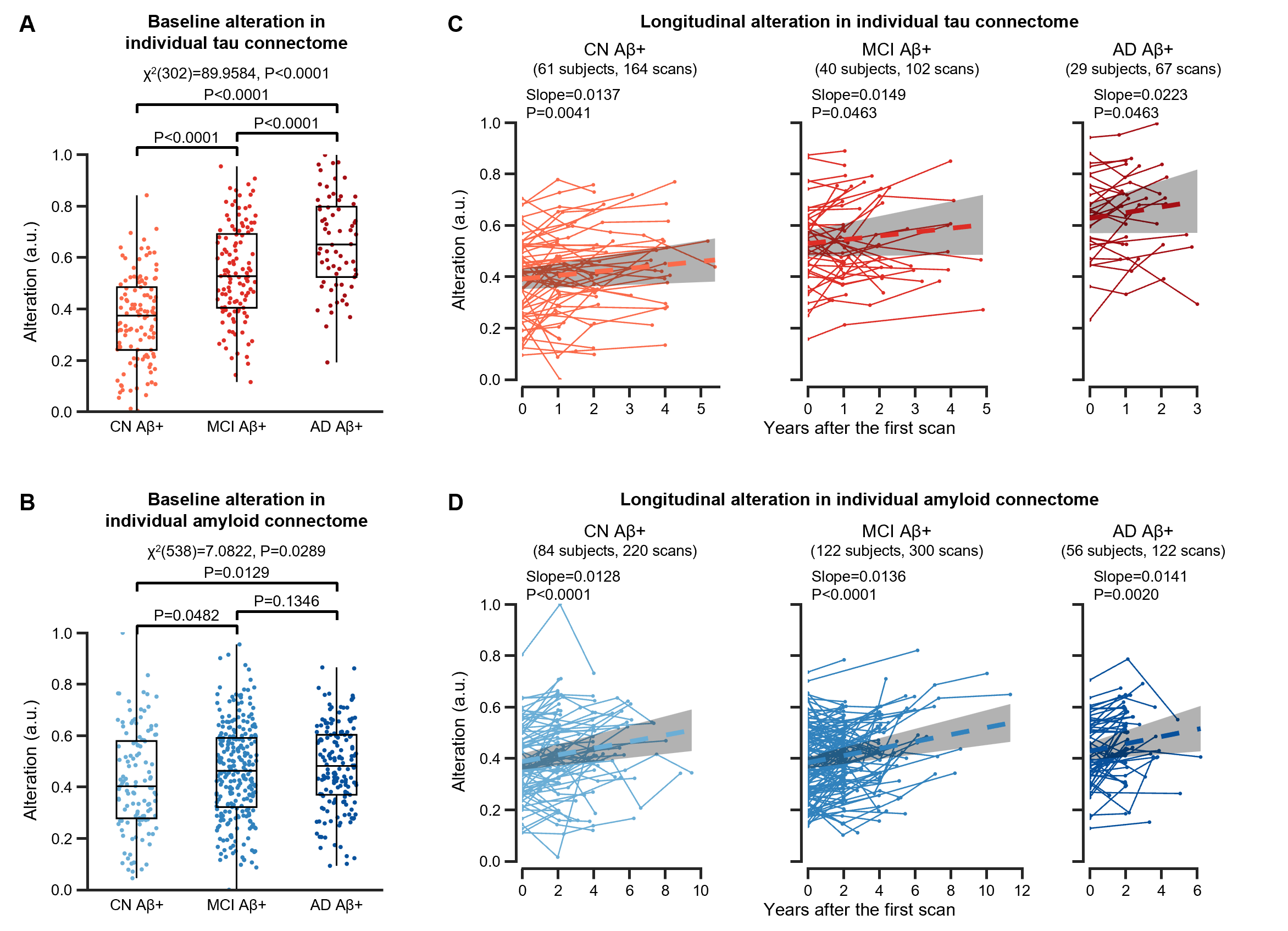
**

**Figure S11. Alterations in individual molecular connectomes increase across the AD continuum and over time when using the Automated Anatomical Labeling (AAL2) atlas of 86 brain regions.** (**A** and **B**), Baseline alterations in individual tau (**A**) and amyloid (**B**) connectomes across the AD continuum. Each dot represents one subject. Box plots depict the interquartile range and the median value of the distribution. Whiskers extend to the nearest data points ±1.5-times the interquartile range. The effects of diagnostic group (CN Aβ+, MCI Aβ+, and AD Aβ+) on individual molecular connectomes were examined by Kruskal-Wallis test followed by post hoc Wilcoxon rank-sum tests. The significance levels of the Kruskal-Wallis test and post hoc Wilcoxon rank-sum tests were evaluated through 10,000 permutations and were corrected for FDR. (**C** and **D**), Longitudinal alterations in individual tau (**C**) and amyloid (**D**) connectomes across the AD continuum. Each dot represents one PET scan. Each thin line connects PET scans from one subject. Bold dashed line and grey shading represent the fitted line and its 95% confidence intervals from the linear mixed-effects model. P values were extracted from the linear mixed-effects model and were corrected for FDR. CN, cognitively normal; MCI, mild cognitive impairment; AD, Alzheimer’s disease; Aβ+, amyloid-β positive; a.u. arbitrary unit. Compare with Figure 3 in the main manuscript.


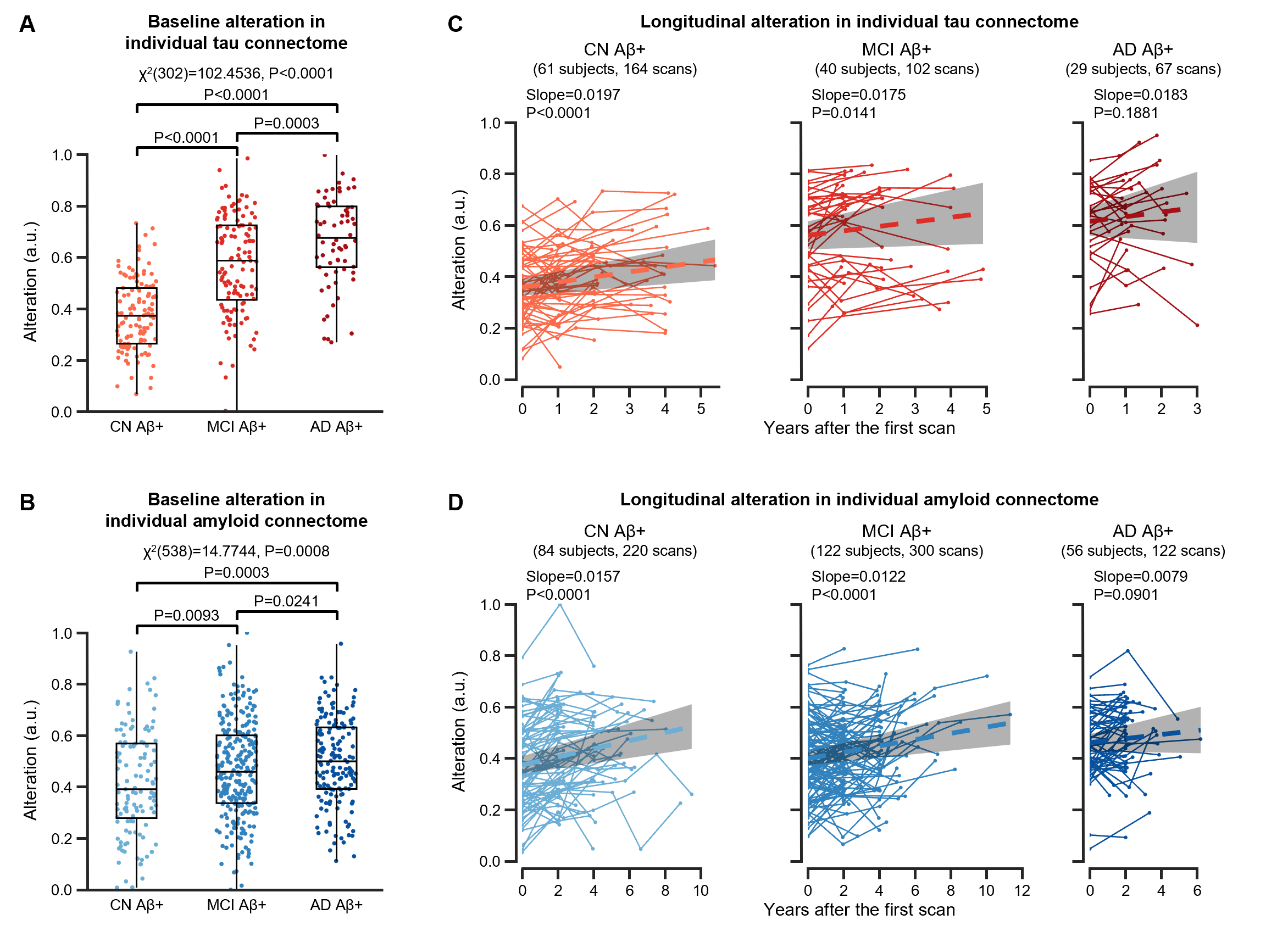


**Figure S12. Alterations in individual molecular connectomes increase across the AD continuum and over time when using the Hammersmith atlas of 50 brain regions.** (**A** and **B**), Baseline alterations in individual tau (**A**) and amyloid (**B**) connectomes across the AD continuum. Each dot represents one subject. Box plots depict the interquartile range and the median value of the distribution. Whiskers extend to the nearest data points ±1.5-times the interquartile range. The effects of diagnostic group (CN Aβ+, MCI Aβ+, and AD Aβ+) on individual molecular connectomes were examined by Kruskal-Wallis test followed by post hoc Wilcoxon rank-sum tests. The significance levels of the Kruskal-Wallis test and post hoc Wilcoxon rank-sum tests were evaluated through 10,000 permutations and were corrected for FDR. (**C** and **D**), Longitudinal alterations in individual tau (**C**) and amyloid (**D**) connectomes across the AD continuum. Each dot represents one PET scan. Each thin line connects PET scans from one subject. Bold dashed line and grey shading represent the fitted line and its 95% confidence intervals from the linear mixed-effects model. P values were extracted from the linear mixed-effects model and were corrected for FDR. CN, cognitively normal; MCI, mild cognitive impairment; AD, Alzheimer’s disease; Aβ+, amyloid-β positive; a.u. arbitrary unit. Compare with Figure 3 in the main manuscript.
